# Integrated network analysis reveals potentially novel molecular mechanisms and therapeutic targets of refractory epilepsies

**DOI:** 10.1371/journal.pone.0174964

**Published:** 2017-04-07

**Authors:** Hongwei Chu, Pin Sun, Jiahui Yin, Guangming Liu, Yiwei Wang, Pengyao Zhao, Yizhun Zhu, Xiaohan Yang, Tiezheng Zheng, Xuezhong Zhou, Weilin Jin, Changkai Sun

**Affiliations:** 1 Department of Biomedical Engineering, Faculty of Electronic Information and Electrical Engineering, Dalian University of Technology, Dalian, China; 2 Liaoning Provincial Key Laboratory of Cerebral Diseases, Institute for Brain Disorders, Dalian Medical University, Dalian, China; 3 Shanghai Medical College, Fudan University, Shanghai, China; 4 College of Electronics and Information Engineering, Tongji University, Shanghai, China; 5 School of Computer and Information Technology and Beijing Key Lab of Traffic Data Analysis and Mining, Beijing Jiaotong University, Beijing, China; 6 Department of Pharmacology, School of Pharmacy, Fudan University, Shanghai, China; 7 Institute of Nano Biomedicine and Engineering, Department of Instrument Science and Engineering, Key Lab. for Thin Film and Microfabrication Technology of Ministry of Education, School of Electronic Information and Electronic Engineering, Shanghai Jiao Tong University, Shanghai, China; 8 Research Center for the Control Engineering of Translational Precision Medicine, Dalian University of Technology, Dalian, China; 9 State Key Laboratory of Fine Chemicals, Dalian R&D Center for Stem Cell and Tissue Engineering, Dalian University of Technology, Dalian, China; University of Catanzaro, ITALY

## Abstract

Epilepsy is a complex neurological disorder and a significant health problem. The pathogenesis of epilepsy remains obscure in a significant number of patients and the current treatment options are not adequate in about a third of individuals which were known as refractory epilepsies (RE). Network medicine provides an effective approach for studying the molecular mechanisms underlying complex diseases. Here we integrated 1876 disease-gene associations of RE and located those genes to human protein-protein interaction (PPI) network to obtain 42 significant RE-associated disease modules. The functional analysis of these disease modules showed novel molecular pathological mechanisms of RE, such as the novel enriched pathways (e.g., “presynaptic nicotinic acetylcholine receptors”, “signaling by insulin receptor”). Further analysis on the relationships between current drug targets and the RE-related disease genes showed the rational mechanisms of most antiepileptic drugs. In addition, we detected ten potential novel drug targets (e.g., KCNA1, KCNA4-6, KCNC3, KCND2, KCNMA1, CAMK2G, CACNB4 and GRM1) located in three RE related disease modules, which might provide novel insights into the new drug discovery for RE therapy.

## Introduction

Epilepsy is a collection of brain disorders, which is characterized by repeated, uncontrolled seizures. Seizures are abnormal firing of brain cells which may cause changes in behavior or attention, which affect over 65 million people in the world[1]. The cause of many epilepsy cases remains unknown, although some are known to result from brain injury, stroke, brain tumor, gene mutation, and/or substance abuse disorders [2–4]. Seizure is correlated with the enhancement of glutamate responses mediated by N-methyl-D-aspartate (NMDA) receptor. The NMDA receptors are up-regulated when epilepsy occurs, and the corresponding ion channels are kept open causing the neurons discharge continuously[5]. Studies show that brain’s neural circuits play a key role in controlling the balance between epileptic and antiepileptic factors[6]. Studies also support the pathogenic role of neuroinflammation in RE [7]. Microglia may initiate a cycle of inflammation-induced seizures and seizure-induced inflammation, and microglia-driven epilepsy may be a primary pathogenic process[8]. Antiepileptic drugs (AEDs), such as phenytoin sodium, phenobarbital and felbamate, are the first line treatment for controlling epileptic seizures [9,10], which are divided into three categories according to their mechanisms of efficacy. They work by inhibiting the voltage-gated ion channels, increasing the inhibitory effect of GABA, or inhibiting the conduction of glutamate-mediated excitability [11–14].The AEDs can inhibit the spread of abnormal firing patterns to distant sites, which are required for the expression of behavioral seizure activity [15]. However, about a third of the patients do not respond to AEDs, and they are considered to have medically refractory epilepsies (RE) [16].

Furthermore, to fully address the drug resistance and side effects of AEDs, researchers have tried to investigate the underlying molecular mechanisms of RE to search for novel molecular targets and drugs[17]. Meta-analysis studies on epilepsy have proposed new understanding of the genetic study of epilepsy [18,19]. Furthermore, with the increase of the data sources on phenotype-genotype associations and protein-protein interactions (PPI), network medicine has provided an insightful approach to understand the molecular mechanisms of complex diseases [20–22].

**Fig 1:**
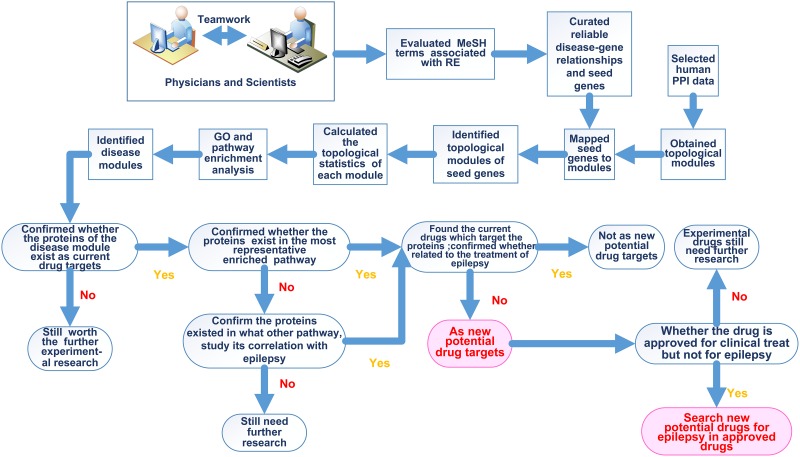
Technical roadmap. The main analytic process was described in frame.

Molecular and genetic studies of epilepsy over the recent decades have produced an impressive list of disease-gene associations, together with the interactome network database, which can be used to investigate the molecular network mechanisms and potential drug targets related to epilepsy[23]. Molecular networks disrupted in epilepsy have been discovered in the brain using an integrated systems-level analysis of brain gene expression data, which may lead to more effective treatments and help us identify new medications for epilepsy[24]. In this study, we curated comprehensive phenotype-genotype associations related to RE to investigate the network module mechanisms and identify the potential drug targets for RE treatment. Here, we follow several main steps to perform the study (Fig 1): 1) Using the RE terms from the Medical Subject Headings (MeSH) terminology, we extracted the seed genes from three databases to construct networks based on the interactions between the proteins they encode; 2) we utilized the a community detection method to obtain disease-related topological PPI modules; 3) Along with the existing AEDs from two databases, we identified three important epilepsy disease modules and ten potential drug targets by conducting gene ontology (GO) and pathway enrichment analysis.

## Materials and methods

### Integrating disease-related genes

The MeSH classification was defined by experts and provided a comprehensive vocabulary across all disease categories, which were systematically organized in a hierarchical tree[25,26]. Based on the 23 MeSH headings, which were extrapolated manually by trained experts, we searched the CoreMine PubMed search engine system[27], the OMIM[28], and the DiseaseConnect[29] databases to extract disease-gene relationships. Moreover, we manually examined and verified disease-gene co-occurrence in the literature contained in the PubMed database, in order to ensure highly accurate relationships. We also checked the HGNC database for the approved name of the gene[30].

### Extraction of PPI network

Detailed human PPI data was downloaded from the STRING 9.1 database[31]. The STRING database is a comprehensive protein-protein interactions (PPIs) data source, which aims to offer a critical assessment and integration of PPIs, including direct (physical) as well as indirect (functional) associations. The various data sources involved experimental, predicted and transferred interactions, together with interactions obtained through text mining. Known and predicted associations are scored and integrated (STRING10.0 has expanded to 9.6 million proteins and 184 million interactions) [31,32]. The weight of each interaction between two proteins was defined by its correlation value. The interactions whose scores> = 700 have high confidence or be considered as a high-quality subset[33]. By filtering the links with the scores> = 700, we finally obtained a high-quality String 9 human subset with 218,157 PPI records and 14,379 distinct proteins.

### Topological module identification

Topological module refers to the subnetwork of one entire network, which has relatively dense links when compared with the links outside of the subnetwork[20]. BGLL, a popular community detection algorithm which is based on modularity evaluation, was used to obtain the topological modules of the whole PPI network. Module size too small or too large is not benefit for the enrichment analysis. The module size limits made us iteratively divided the modules using BGLL, resulting in topological modules that contain between 5 and 400 member nodes [34,35]. The weight of the edges between the modules was calculated using the formula:

$$weight=\frac{c}{a\times b}$$ (1)

where c is the real edge number, a and b represent the number of proteins in each module.

### GO and pathway enrichment analysis

Many online analysis platforms, as well as relevant analysis softwares, are available for GO enrichment analysis [36,37]. In this study, the results of GO enrichment analysis were performed using the BiNGO 2.44 plug-in for the Cytoscape 3.0.2 software[38], according to the significance threshold (p-value<0.05). Application of BiNGO in molecular interaction networks, e.g. protein interaction networks, visualized in Cytoscape is better than other tools. BiNGO can map the dominant themes of the target gene based on GO hierarchy, and it also produce an intuitive and customiazable visual representative results by Cytoscape’s versatile visualization platform [39].

The BiNGO 2.44 plug-in can map the genes to GO terms using a hypergeometric distribution relationship. Then, we can obtain the GO terms with related genes. Through Bonferroni correction to control the false positive rate of analysis, this process will return a p-value. All the results of pathway enrichment analysis were obtained through the online analysis tool KOBAS 2.0[40], according to the significance threshold (p-value<0.05). The KOBAS 2.0 can return the results of six pathway databases (Reactome, KEGG PATHWAY, BioCyc, PANTHER, BioCarta, and PID). We chose the Reactome database in our study because its results were the most comprehensive[41]. By calculating the hypergeometric distribution relationship, we can pick out statistically significant pathways. Through Bonferroni correction to control the false positive rate of analysis, this process will also return a p-value. Network and module visualizations were designed with the help of the visualization software, Gephi [42], while the heat map was created with the use of hemI software.

### Function-based modules similarity

A widely used method in both text mining and biomedical literature to quantify the similarity between two concepts is the cosine similarity of respective vectors [26]. In this study, we applied the cosine similarity method to calculate function-based similarity. We used the equation −*log*(*corrected p* − *value*) to transform p-values to correlation values, since the smaller of the corrected p-value small the bigger of the correlation between modules and functional entities. The similarity between the vectors, A and B, of the two modules, A and B, is calculated as follows:

$$cos\left(A,B\right)=\frac{\sum_{i=1}^{n}A_{i}B_{i}}{\sqrt{\sum_{i=1}^{n}A_{i}^{2}}\sqrt{\sum_{i=1}^{n}B_{i}^{2}}}$$ (2)

where A_i_ and B_i_ are components of vector A and B respectively. The range of functional similarity is zero (no similarity of two modules) to one (two modules have same function), and a larger value for similarity represents more functional similarity between two modules. The combined score equals 0.5*((BP similarity+CC similarity+MF similarity)/3) +0.5*Pathway similarity.

### Shortest paths between drug targets and seed genes

We searched for relevant drugs by searching for disease keywords in the ‘indications’ field of drug information obtained from the DrugBank database, which combines detailed drug data with comprehensive drug-target information[43,44]. Shortest paths, a significant topological quantity, are often used for the analysis of social and biological networks, such as the well-known small world property of many complex networks [26,45,46]. We used the Dijkstra’s algorithm to find the shortest path lengths between epilepsy drug targets and seed genes[47]. To obtain random controls for the target-seed gene, we generated 100 independent randomized samples in the PPI network. Significant difference was calculated statistically using Student’s test.

## Results

### MeSH heading of RE subtypes

Using epilepsy as the keyword, MeSH terms related to RE were extracted and evaluated from the MeSH vocabulary. As shown in Table 1, 23 MeSH headings were identified, which were typical and significant disease phenotypes, such as lissencephaly and myoclonic epilepsy (“Epilepsies, Myoclonic” as the MeSH heading of Dravet syndrome which was one of the best experimentally studied drug resistant primary epilepsy syndromes). The included syndromes are basically epilepsy syndrome. Individual syndrome belongs to always accompanied by epileptic seizures. Targets and pathways cross associated with epilepsy syndrome, but not a typical epilepsy syndrome. Anti-N-Methyl-D-Aspartate Receptor Encephalitis was known not long time, but Alexander Disease was known for a long time.

**Table 1:** Twenty three MeSH headings of RE subtypes.

NO.	MeSH heading	Unique ID	NO.	MeSH heading	Unique ID
1	Alexander Disease	D038261	13	Malformations of Cortical Development	D054220
2	Anti-N-Methyl-D-Aspartate Receptor Encephalitis	D060426	14	MELAS Syndrome	D017241
3	Classical Lissencephalies and Subcortical Band Heterotopias	D054221	15	MERRF Syndrome	D017243
4	Epilepsies, Myoclonic	D004831	16	Mitochondrial Encephalomyopathies	D017237
5	Epilepsy, Absence	D004832	17	Myoclonic Epilepsies, Progressive	D020191
6	Epilepsy, Frontal Lobe	D017034	18	Myoclonic Epilepsy, Juvenile	D020190
7	Epilepsy, Temporal Lobe	D004833	19	Neuronal Ceroid-Lipofuscinoses	D009472
8	Fragile X Syndrome	D005600	20	Rett Syndrome	D015518
9	Lafora Disease	D020192	21	Spasms, Infantile	D013036
10	Landau-Kleffner Syndrome	D018887	22	Tuberous Sclerosis	D014402
11	Leigh Disease	D007888	23	Unverricht-Lundborg Syndrome	D020194
12	Lissencephaly	D054082			

The MeSH headings which were listed in this manuscript all were according to the consensus of International League Against Epilepsy (ILAE) [48,49]. In this study, we focused on several key MeSH headings, such as Dravet syndrome, Lafora disease and refractory temporal epilepsy. Considering the complexity of epilepsy syndrome, other MeSH headings were all important related disorders or diseases (e.g. curable temporal epilepsy), which were used for comparable purposes. We hope to further understand and identify the new general or individual targets of refractory epilepsies through the comparable and extended research.

Twenty-three MeSH headings were integrated in order to make comparisons to understand the reliability of target screening for correlation with disease phenotype.

### RE associated genes and their functional characteristics

**Fig 2:**
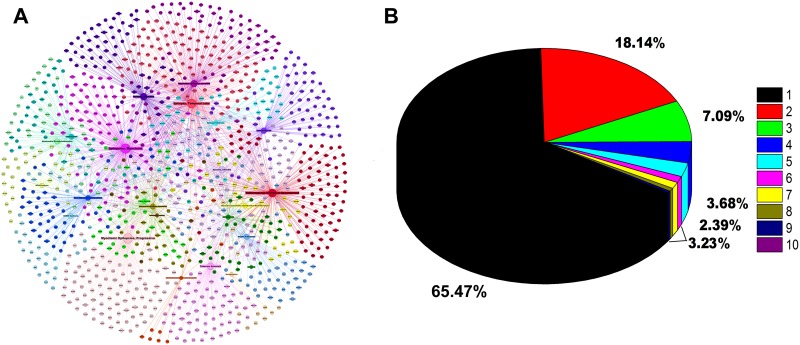
Refractory Epilepsies (RE) disease-gene network and relationships statistics. (A) Disease-gene network showing the relationships of 1,086 genes corresponding to 23 MeSH Headings. The long label big nodes denote diseases in MeSH terminologies; the small nodes denote genes. The size of the node is positively related to the number of linked genes. Different colors represent different diseases. Genes located in the center of the network are associated with several diseases (e.g. SCN1A, KCNQ2). Genes located at the periphery of the network are associated with a single disease (e.g. CHRNA5, CHRNB4). (B) The gene-related MeSH heading numbers are distributed from 1 to 10, with different numbers for different colors. The total number of seed genes is 1086, of which 711 (accounting for 65.47%) are only associated with one disease subtype (MeSH heading).

After filtering the relationships with significant correlations (i.e. p-value < 0.05), total 3,219 disease-gene relationships were obtained with the corresponding number of occurrences in the CoreMine PubMed search engine system[27]. All literatures related to the 3,219 relationships in the PubMed database were analyzed and only 1,852 disease-gene relationships with 1,065 distinct genes were identified. There were additional 24 disease-gene relationships with 21 distinct genes, which were not included in the CoreMine data sources by checking the Online Mendelian Inheritance in Man (OMIM)[28] and DiseaseConnect[29] databases. All together we finally obtain 1,086 RE-related genes for further analysis. To further validate the reliability of the genes, we also conducted the external validation analysis of epilepsy disease-gene associations using the latest data from the high-quality genotype-phenotype associations of Human Phenotype Ontology (HPO) database [50]. Using “seizure or seizures” as keywords, we obtained 662 genes from HPO disease-gene associations that are related to disease phenotypes with seizure manifestations. There were 215 (215/662 = 32.5%) genes were overlapped with the 1086 RE-related genes, which has 5.70-folds over random expectations (p-value<9.39E-100; binomial test). This result indicates that our curated disease-gene associations have significant overlap with the gene list associated with seizure phenotypes and thus further means that our data is reliable for further analysis. The disease-gene association network with 1876 links was visualized accordingly and it showed clearly that the 23 RE related disease phenotypes (in terms of MeSH headings) both have their own distinct genes and many shared genes among them (Fig 2A). In addition, we found that most (711/1086 = 65.47%) of the genes related to just one disease subtype (Fig 2B). However, substantial ratio (~35%) of the genes is associated with multiple disease subtypes. For example, SCN1A associates with 10 disease subtypes, while the four genes: KCNQ2, NHLRC1, PCDH19 and SCN1B associate with 9 subtypes.

To obtain the functional descriptions of RE associated genes, we performed the enrichment analysis of the GO and pathways of these genes[51]. GO contains three hierarchically structured vocabularies which describe gene products in terms of their connected biological processes (BP), cellular components (CC) and molecular functions (MF)[39]. We obtained 1014 enriched BP terms, 214 CC terms, and 176 MF terms (S1–S3 Tables) of the RE associated genes. For example, the specific level enriched GO terms that characterize the functions of these genes include, BP terms: ion transport (fifth level), generation of neurons (seventh level), and regulation of transmission of nerve impulse (sixth level); CC terms: neuron projection (fifth level), ion channel complex (fifth level), and postsynaptic membrane (fifth level); and MF terms: excitatory extracellular ligand-gated ion channel activity (eighth level), neurotransmitter receptor activity (fifth level), and NADH dehydrogenase (ubiquinone) activity (seventh level). This indicates that the well-known molecular mechanisms of RE that involve nervous system development and intercellular signaling transduction [52–54].

In addition, it also showed the potential novel mechanisms of RE, which included the enriched GO terms, such as “regulation of programmed cell death” (sixth level), “apoptotic process” (sixth level), “response to ethanol” (sixth level) and “response to nicotine” (sixth level). Recent study suggested that neuronal death seems closely linked to epileptogenesis, but the effect of seizures on neuronal death and the role of seizure-induced neuronal death in acquired epileptogenesis need further investigation[55,56]. Similarly, cigarette smoking and alcohol misuse were considered as the behavioral risk factors associated with epilepsy [57,58], which indicated that the mechanism for nicotine-induced and alcohol-induced seizure would be valuable for further investigation.

Furthermore, we identified 55 enriched pathways (corrected p-value<0.05) in the Reactome database (S4 Table), in which most pathways were consistent with the results of GO enrichment analysis. For example, the pathways, such as “mTOR signaling pathway”, “highly calcium permeable postsynaptic nicotinic acetylcholine receptors”, “potassium Channels”, “GABA A receptor activation”, “unblocking of NMDA receptor” and “glutamate binding and activation”, have close relationships to RE. It is well established that the abnormal activities of ion channels, neurotransmitters, and NMDA receptors lead to synaptic euphoria, which may also be involved in the onset of epileptic seizures[59]. Furthermore, the citric acid cycle (TCA) and respiratory electron transport, which had important roles in RE development, were also identified to be enriched pathways. It is well-known that epilepsy has a strong genetic background with the combined risk related to hundreds or thousands of genes. Identifying the gene network or pathways that underlying epilepsy is important for detection of new targets for anti-epilepsy medications. Other enriched pathways, such as “presynaptic nicotinic acetylcholine receptors”, “signaling by insulin receptor”, may play a role in the development of RE and its therapeutic responses [60–62].

### Disease modules that reveal the underlying molecular mechanisms of RE

Disease associated genes are not distributed randomly on the molecular interaction network and they tend to work together in similar biological modules or pathways[63]. To investigate the underlying molecular modular mechanisms of RE, we obtained high quality human PPI network (filtered by a significant score > = 700) from the STRING 9.1 database [64,65]. Finally, using a widely used community detection algorithm (see Methods), we identified 314 topological modules covering 13,733 proteins and 136,800 edges in the human PPI network. To obtain the RE disease modules, we calculated the overlap ratio (in terms of Relative Risk, RR)) between RE associated genes and the genes of each topological module. There were 921 (921/1086 = 84.8%) associated genes distributed in 185 (185/314 = 58.9%) modules, which contained 12,084 (12,084/13,733 = 88.0%) genes/proteins in total.

We combined two filtering conditions to obtain the significant associated modules for RE disorders. Firstly, we calculated the correlation between 185 modules and RE disorders by Chi-Square test (with Bonferroni correction) and finally we found that there are five modules with corrected p-value<0.01, in which module M37 is the last top ranked module with corrected p-value ~3.67e-4 (S5 Table). It suggested that those five modules may have a significant correlation with RE. Interestingly, all proteins (i.e., GRM1-8) of module M145 were seed genes of RE, which form a dense network corresponding to the glutamate receptor complex. Module M37 has 305 genes and overlapped 45 (45/305 = 14.8%) seed genes with RE associated gene list and thus has a RR value 1.94. In fact, there are many modules with less number of genes, which although have not passed the most conserved type of multiple testing correction method, have much larger RR values over the RR value of M37. Therefore, finally we consider the RR value of M37 as the threshold to filter the significant associated modules of RE disorders, which include 42 modules (S6 Table). It is these 42 primary modules are used for further analysis.

**Fig 3:**
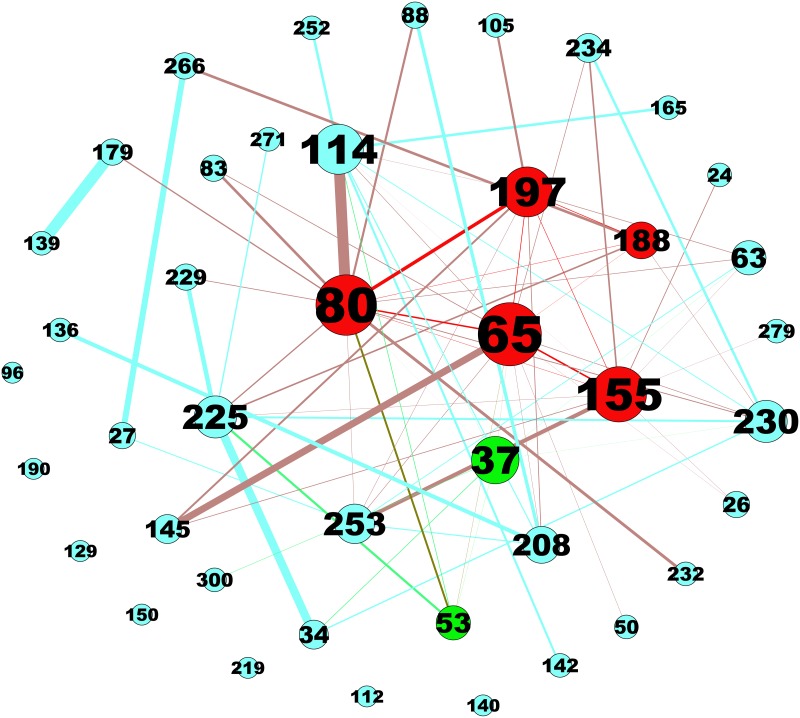
Interactions between 42 topological modules. Red nodes: seed genes of modules > = 10; blue nodes: seed genes of modules < 10; green nodes: extensional modules. Node size corresponds to degree of module. The thickness of the edge is proportional to the weight, and the weight corresponds to strength of interactions.

To distinguish the core modules from those 42 significant modules, we constructed a network with nodes representing the modules and links representing shared PPI interactions between them (Fig 3). It showed that there are six modules (M37, M65, M80, M114, M155, and M197) with significantly higher degrees than that of the other modules (Table 2), which means that these six modules are the central modules underlying RE molecular pathologies[66].

**Table 2:** Top one enriched GO terms in the main ten modules.

Module	Size	Degree	BP		CC		MF	
			GO term	p-value	GO term	p-value	GO term	p-value
M37	305	10	Oxidative phosphorylation	< 1E-100	Mitochondrial membrane part	<1 E-100	Hydrogen ion transmembrane transporter activity	< 1E-100
M65	184	16	Ion transport	2.29E-43	Calcium channel complex	9.34E-47	Gated channel activity	3.87E-53
M80	52	15	Regulation of TOR signaling cascade	8.69E-13	Cytosol	1.31E-07	Protein serine/threonine kinase activity	4.76E-05
M114	53	11	Regulation of fatty acid oxidation	4.68E-22	AMP-activated protein kinase complex	1.90E-11	Protein serine/threonine kinase activity	2.68E-15
M145	7	3	Negative regulation of cyclase activity	8.16E-10	Integral to plasma membrane	2.81E-07	Glutamate receptor activity	8.27E-19
M155	141	13	Ion transport	4.02E-69	Ion channel complex	6.63E-83	Gated channel activity	6.01E-98
M188	43	6	Synaptic transmission, cholinergic	1.10E-28	Nicotinic acetylcholine-gated receptor-channel complex	5.90E-38	Nicotinic acetylcholine-activated cation-selective channel activity	9.95E-35
M197	50	11	Membrane invagination	1.35E-09	Coated pit	5.83E-07	Lipoprotein binding	1.68E-09
M208	34	6	Muscle organ development	3.62E-09	Dystrophin-associated glycoprotein complex	1.09E-42	Calcium ion binding	8.94E-04
M230	45	8	Protein localization	6.44E-15	Endosome	3.62E-29		

Size means the number of proteins in each module. Degree corresponds to number of edges connected with module. The function of a module is very similar to those of the proteins in it. The p-value is negatively related to the enrichment.

We further examined the enriched GO terms of the 42 modules (Table 2, S7 Table), in which we only list the top enriched specific GO terms. For example, the three modules, namely M37, M155 and M65, have enriched terms with lowest p-values, which include the BP terms: oxidative phosphorylation, ion transport, and synaptic transmission; the CC terms: mitochondrial inner membrane, channel complex, and synapse part; and the MF terms: hydrogen ion transmembrane transporter activity, ion channel activity, and gated channel activity.

**Fig 4:**
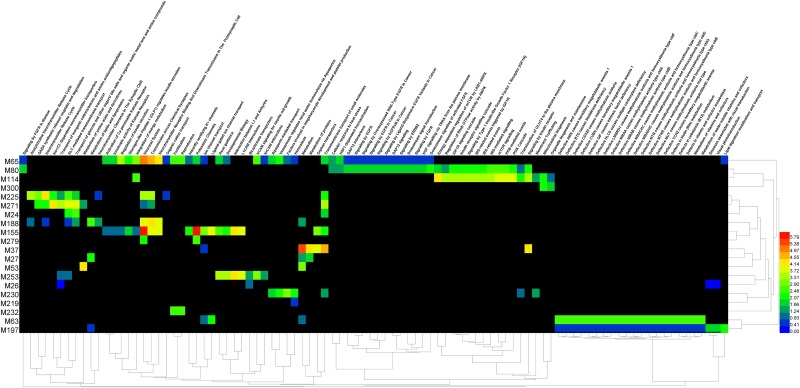
Heat map of 94 common pathways corresponding to enrichment in 20 modules. X-axis: pathway; Y-axis: module. Color key from blue to red is representative of low to high enrichment (p value from large to small). If the cross of module and pathway form a black cell, it refers to that the pathway in the module is not enriched.

Most modules (except module M139 and M150) have their enriched pathways using six pathway databases in KOBAS 2.0. However, we only obtained 317 enriched pathways (corrected p-value<0.05) for 28 modules using the Reactome database. The 317 pathways were classified into two types according to whether it was located in a single module or multiple modules. We call the former as “individual pathway” and the latter as “common pathway” for RE. Statistical analysis yielded 223 individual pathways located on 27 modules, and 94 common pathways distributed across 20 modules. We generated a heat map to display the 94 common pathways corresponding to their enrichments in 20 modules (Fig 4). It showed that the common enriched pathways also referred to the branch of transmission across chemical synapses, neurotransmitter receptor binding and downstream transmission in the postsynaptic cell. Neurotransmitter secretion is triggered by the influx of Ca^2+^ through voltage-gated channels, which gives rise to a transient increase in Ca^2+^ concentration within the presynaptic terminal. Module M37, M65 and M155 had significant enrichment pathways (red color) among the 20 modules. For module M37, the highly enriched individual pathway was “Respiratory electron transport, ATP synthesis by chemiosmotic coupling, and heat production by uncoupling proteins”. Module M65 was closely related to the “Activation of NMDA receptor upon glutamate binding and postsynaptic events” “Glutamate Binding, Activation of AMPA Receptors and Synaptic Plasticity” “Trafficking of AMPA receptors” and “Unblocking of NMDA receptor, glutamate binding and activation”. Module M155 was most related to the “activation of voltage-gated potassium channels”, “GABA receptor activation”. NMDA receptor-mediated signal transduction is critical for synaptic plasticity. In acute and chronic seizures, a selective NMDA receptor antagonist has broad clinical application prospects[5]. It has been shown that CaV3.2 channels regulate NMDA receptor mediated transmission and subsequent NMDA receptor dependent plasticity of AMPA-R-mediated transmission[67].

To further detect the functional similarity between two module pairs, the vectors corresponding to each module were constructed using their GO terms and pathways as features, and the cosine similarity of the module vectors was calculated to quantify the similarity between the two modules pairs. A combined score was then used to comprehensively evaluate the functional similarity between these modules. The combined scores of the top seven module pairs are shown in Table 3 (S8 Table). As we can see, module M65 and M155 exhibited the most functional similarity among the 42 modules.

**Table 3:** Combined score of top seven pair of modules.

Module1	Module2	GO-BP	GO-MF	GO-CC	Pathway	Combined score
M65	M155	0.615	0.814	0.557	0.364	0.513
M279	M155	0.00572	0.323	0.0173	0.753	0.434
M80	M114	0.0966	0.644	0.240	0.515	0.421
M65	M188	0.358	0.490	0.356	0.268	0.335
M271	M225	0.446	0.323	0.175	0.332	0.323
M271	M24	0.0747	0.347	0.424	0.317	0.299
M188	M155	0.306	0.417	0.386	0.178	0.274

Only 36 (36/42 = 85.7%) modules that had functional similarity were classified into five clusters using the community detection algorithm in Gephi software. Each cluster corresponds well to the RE MeSH headings related to the PPI topological modules (RR>1.0). These allocations were based on the observation that genes causing similar diseases tend to link to each other in the interactome [68,69]. The patients with similar clinical manifestations may have different underlying disease mechanisms. Moreover, gene products linked to the same phenotype have a strong tendency to interact with each other and to cluster in the same network neighborhood[70]. It showed that most of the MeSH headings map multi-modules, especially Myoclonic epilepsies (Table 4).

**Table 4:** The number of modules corresponded to MeSH headings.

MeSH heading	Number of Module	Module	MeSH heading	Number of Module	Module
Alexander Disease	1	M80	Fragile X Syndrome	5	M88,M145,M230
					M234,M271
Leigh Disease	1	M37	Myoclonic Epilepsy, Juvenile	6	M83,M105,M140
					M145,M155,M266
MERRF Syndrome	1	M208	Epilepsy, Temporal Lobe	7	M63,M105,M145,M150
					M155,M225,M232
Mitochondrial Encephalomyopathies	1	M300	Spasms, Infantile	7	M53,M63,M80,M139
					M155,M208,M253
Epilepsy, Frontal Lobe	2	M188,M266	Epilepsy, Absence	8	M65,M105,M145,M155
					M188,M266,M271,M279
MELAS Syndrome	2	M37,M300	Rett Syndrome	8	M26,M50,M53,M145
					M225,M234,M252,M271
Classical Lissencephalies and Subcortical Band Heterotopias	3	M34,M80,M188	Tuberous Sclerosis	8	M24,M80,M83,M114
					M129,M179,M230,M234
Unverricht-Lundborg Syndrome	3	M27,M63,M129	Malformations of Cortical Development	9	M34,M65,M80,M88,M112
					M145,M165,M208,M225
Lafora Disease	4	M27,M114,M155,M230	Myoclonic Epilepsies, Progressive	9	M27,M53,M114,M129,M150
					M155,M190,M225,M232
Lissencephaly	4	M80,M114,M197,M208	Epilepsies, Myoclonic	12	M63,M105,M112,M136,M139,M142
					M155,M179,M188,M219,M266,M300
Neuronal Ceroid-Lipofuscinoses	4	M27,M96,M188,M229			

We have further investigated the associated modules corresponded to each MeSH headings and actually we found that there are distinct modules associated to some specific RE MeSH headings (Table 4, e.g. module M197 for Lissencephaly, M136 for Myoclonic Epilepsies), which means that some RE disease subtypes have their corresponding distinct molecular mechanisms. Furthermore, we found that different MeSH headings have substantial number of shared modules, which makes some modules such as M155 and M80 associated to multiple MeSH headings. This suggested that these modules (e.g.M155) would be the common underlying molecular network of different RE subtypes. These indications could be further confirmed from the results of the shared genes (using Jaccard similarity) between each RE MeSH headings (S9 Table). We found that most RE MeSH headings have some degree of shared genes between each other.

Moreover, frontal lobe epilepsy and temporal lobe epilepsy have very different gene distributions within their modules. Frontal lobe epilepsy with 49 associated genes correlates to two modules when RR>1.0, while temporal lobe epilepsy with 185 associated genes correlates to seven modules when RR>1.0. The therapeutic effect of different brain regions affected by disease varies considerably, which can, to some extent, be demonstrated from the difference in module distribution. Although the module distribution is disparate, there were functional similarities between the modules, which might indicate shared similar molecular pathologies between them.

### The underlying mechanisms of AEDs from the perspective of drug-target-gene interactions

**Fig 5:**
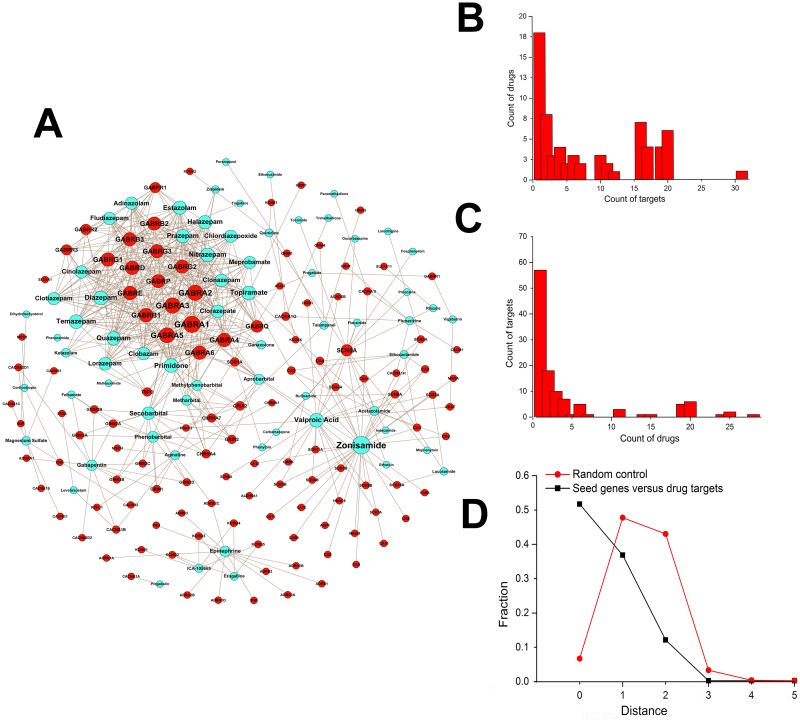
Refractory Epilepsies (RE) existing drug-target network, statistical analysis about drug- target and minimum shortest path analysis. (A) Drug-target network. Blue nodes: drugs; red nodes: targets. The network was generated by using the known associations between drugs and their targets from the DrugBank and Sider databases. The size of each drug (target) node is proportional to the number of targets that the drug has (the number of drugs targeting the protein), respectively. A link is placed between a drug node and a target node if the protein is a known target of that drug. One drug can target multiple proteins, and one protein can be targeted by multiple drugs. (B) Distribution of drugs with respect to the number of their targets; (C) Distribution of targets with respect to the number of effective drugs. (D) Distribution of the shortest distances of the actual data and random protein groups.

Related antiepileptic terms, such as anticonvulsant, antiseizure, and antiepileptic, were used to obtain the names of current AEDs from the DrugBank and Sider databases [43]. Finally, 68 drugs and their 119 corresponding targets were identified and obtained (Fig 5A). Most drugs target only a few proteins, but some, like Zonisamide and Diazepam, have many targets. It has been suggested that the eight GABA receptors such as GABRAx, GABRB1, and GABRD are targeted by more than 20 drugs each, and were the top eight proteins targeted by these drugs. The top seven drugs with most targets included Diazepam, Nitrazepam, and Primidone. They all had more than 20 target proteins, which were GABA receptors, and had significant overlap between each other. The distribution of drugs per target and the distribution of targets per drug are shown in Fig 5B and 5C. There were 18 (18/68 = 26.5%) drugs with only one target, and 57 (57/119 = 47.9%) target proteins targeted by only a single antiepileptic drug. This suggested that new drugs tend to bind known target proteins, which had shown obvious limitations in the treatment of epilepsy. The 119 drug targets were mapped to 13,733 proteins, with an overlap for 105 (105/119 = 88.2%) drug targets. The minimum shortest distances between 105 drug targets and 921 seed genes were measured to analyze their interaction path lengths. The actual mechanism through which a drug acts may be unknown, however, the number of molecular steps between a drug target and the corresponding disease cause can be estimated by the shortest distance [44]. A clear enrichment was observed in regions with the lower shortest distances, in comparison with randomized gene groups of similar size and pairing (Fig 5D; p-value = 3.63E-126, t-test), showing the high direct-target effect of current AEDs. Moreover, this provided evidence that most AEDs directly corresponding molecular markers that aid the understanding of the cause of RE.

Drugs do not target diseases equally, but are clearly enriched in some modules[44]. To quantify this effect and investigate the distribution of drug targets, the shortest path length between the 105 drug targets and the 314 modules was calculated. The results showed that M155 had the greatest number of targets (19 direct targets and 8 targets as the first neighbors of seed nodes), followed by M65 (16 direct targets and 8 targets as the first neighbors of seed nodes). The other modules had <7 targets (S10 Table).

The most important modules that represented the greatest correlation were selected as the modules that included more drugs that are available. To understand the degree of a module in the RE drug discovery, it would be relevant to normalize the number of drugs by the number of proteins of each module. The number of drugs corresponding to each module was determined statistically (S10 Table). M155 had the greatest number of drugs (48 drugs), followed by M65 (20 drugs). Module M95 was not in the primary 42 modules, although it had 21 drugs. The other modules had <11 drugs. The results indicated that modules M155 and M65 had the greatest potential with RE pharmacology and were considered the most important modules for drug discovery. The two modules had the proteins, such as KCNA2, GABRA1, and GRIN2A, which had been recently be discovered as novel epilepsy genes [71].

### Novel potential drug targets detected in RE disease modules

**Fig 6:**
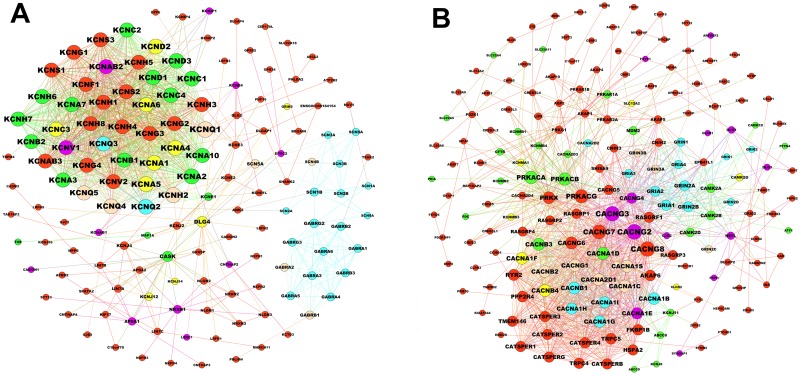
Disease module network. (A) Protein classification network of module M155. The protein nodes are color-coded. Light pink nodes: currently known epilepsy drug targets which are not seed genes; green nodes: currently known drug targets, not specific for epilepsy, which are not seed genes; red nodes: currently unknown drug targets which are also not seed genes; blue nodes: currently known epilepsy drug targets which are also seed genes; yellow nodes: currently known drug targets—seed genes, not specific for epilepsy; purple nodes: currently unknown drug targets—seed genes, not specific for epilepsy. (B) Protein classification network of module M65. The node coloring is the same as that of module M155.

A disease module, a local neighborhood of the interactome whose perturbation is associated with epilepsy, can be mechanistically linked to a particular disease phenotype[70,72]. The precise identification of such disease modules could help with the elucidation of molecular mechanisms, identification of new disease genes, and related signaling pathways, and aid with rational drug target identification[69]. Although module M37 had the greatest number of seed genes, there were no available AEDs for module M37, which had only 10 (10/305 = 3.3%) proteins targeted by drugs for the treatment of non-epileptic disorders. Module M155 had the most seed genes and the greatest number of drugs when RR> = 2.0. From the aforementioned functional analysis, Module M155 can be identified as one of the most important modules for drug discovery. The most enriched terms are ion transport for BP, ion channel complex for CC, gated channel activity for MF, and the enriched pathway is the activation of voltage gated potassium channels, which is the only pathway under voltage gated potassium channels in the Reactome database. Potassium channels are important determinants of seizure susceptibility by modulating the electrical activity of neuronal and non-neuronal cells in the brain. The 141 proteins of module M155 were classified into six types on the basis of whether seed gene or drug target (Fig 6A) and there were 43(43/141 = 30.5%) proteins located in the pathway of activation of voltage-gated potassium channels.

In order to be considered as a novel drug target for epilepsy, the protein still needs to fulfill the following two conditions: 1) no known anti-epilepsy drugs target this protein; 2) The target should exist in the most enriched pathways related to epilepsy. Considering these two conditions, we finally identified six proteins (i.e. KCNA1, KCNA4, KCNA5, KCNA6, KCNC3 and KCND2) from M155 as the potential drug targets (Fig 6A).

The aforementioned functional similarity analysis revealed that module M155 and M65 had the largest correlations. Drugs distribution showed that module M65 had the second highest number of drugs when RR > = 2.0. Similarly, we, obtained four proteins (i.e. KCNMA1, CAMK2G, CACNB4 and KCNMB3) from module M65 as potential drug targets (Fig 6B), and one (GRM1) from module M145 (actually M145 is a protein complex). The expression of 11 proteins at the tissue and cellular levels are shown in Table 5. Specific gene mutations occurring exclusivelyin the brain can lead to RE, which was identified as one of the causes of RE [4]. Hippocampal sclerosis and dysplasia of the cerebral cortex have been considered vitally important pathogenic factors of RE[73,74]. KCNMB3 is not expressed in the cerebral cortex and hippocampus; therefore, it could not be considered as a candidate drug target. In conclusion, ten potential drug targets were finally identified for RE in this study.

**Table 5:** Expression of potential drug targets in tissues and cells.

Tissue	Cerebral Cortex				Hippocampus	
Cell	Neuronal cells	Glial cells	Endothelial cells	Neuropil	Neuronal cells	Glial cells
**M155**	KCNA1	KCNA5	KCNA1	KCNA1	KCNA1	KCNA6
	KCNA4	KCNA6	KCNA4	KCNA4	KCNA4	
	KCNA5		KCNA5	KCNA5	KCNA6	
	KCNA6		KCNA6	KCNA6	KCNC3	
	KCNC3		KCND2	KCNC3		
				KCND2		
**M65**	CAMK2G	CAMK2G	CACNB4	KCNMA1	CAMK2G	CACNB4
	CACNB4	CACNB4		CAMK2G	CACNB4	
				CACNB4		
**M145**	GRM1	GRM1		GRM1	GRM1	GRM1

The target tissue information can be searched in the human protein atlas database.

## Discussion

In this study, network medicine approaches were used to integrate the data from multiple databases to investigate the molecular mechanisms and possible drug targets of RE. Total 1086 genes associated with RE were curated after validating each disease-gene relationships from medical literature. This gene list might be the most comprehensive phenotype-genotype association data repository for studying RE molecular mechanisms. Forty two primary disease-related gene networks (modules) were constructed and selected based on the interaction information of gene-encoded proteins in which about 35 modules had significant interactions and functional similarity between each other. These architectures of interaction module indicated the complicated molecular mechanisms of RE and the possible pharmacological targets of RE personalized treatment. The detected RE-related modules provided a potential subtyping of RE from molecular network perspectives. For example, three significant core modules (M155, M65 and M145) may contain potential drug targets. The protein components of these three modules were consistent with the current understanding of pathological mechanisms and pharmacology of epilepsy. Besides, the significant enriched pathways, for example, “GABA A receptor activation”, “unblocking of NMDA receptor, glutamate binding and activation”, are likely the main biological mechanisms of RE and deliver highly insightful information for its drug discovery.

The ten potential targets for antiepileptic medications could be classified into two groups including the ion channel (potassium and calcium channel) and glutamate receptors. Consistently, the KCNAs, components of potassium channel, were detected as the potential drug target for RE in this study. Ezogabine or retigabine was an antiepileptic drug that reduces neuronal excitability by enhancing the activity of the KCNQ potassium channels; KCNAs (e.g. KCNA1 and KCNA6) and KCNQ2 are associated with peripheral nerve hyperexcitability in humans[75]. Based on the aforementioned network analysis, KCNA1 and KCNA6 belong to M155 which is the most important module for epilepsy. Both KCNA1 and KCNA6 involved in cell signal transduction. Taken together, KCNA1 and KCNA6 might have the potential to become the best novel targets for antiepileptic drug discovery. CACNB4 could directly couple electrical activity to gene expression, which was responsible for a type of juvenile myoclonic epilepsy[76]. Recent studies had shown that ganoderma lucidum polysaccharides may inhibit calcium overload and promote CaMK II α expression to protect epileptic neurons[77].

However, we searched the drugs for these ten potential targets from the Drugbank database, and found that most of the drugs were general anesthetics (Enflurane, Methoxyflurane, and Sevoflurane, etc.) or potassium channels blockers (Dalfampridine, Amitriptyline). Although the ten potential drug targets that were identified in this study have approved drugs but they are not antiepilepsy drugs. This means that these ten drug targets might be the novel targets for epilepsy treatment since they located in the significant associated modules of RE disorders. In addition, these approved drugs for other diseases might have the opportunity to be repurposed for RE treatment as well. As a matter of fact, six of the ten potential targets (i.e. KCNA1, KCNA4, KCNA5, KCNA6, KCNC3 and KCND2) have been already used in clinical practice. These proteins are the targets of Dalfampridine, which is a potassium channel blocker used to help multiple sclerosis patients walk [78]. Dalfampridine is a neurofunctional modifier and the first drug that was specifically approved to help with mobility in these patients. However, Dalfampridine may cause seizure as its serious side effect [79,80]. This meant that there were still no particular drugs aiming for these ten potential targets to treat epilepsy. Nevertheless, our results indicate that further studies to elucidate the precise intervention of these targets might be valuable for novel drug development of RE.

Our results showed that the curated RE-related 1086 genes were enriched in the nicotine addiction pathway. It reported that nicotine addiction could cause seizures in human subjects and reduction in the activity of the glutamate transporter type 3, leading to a decrease in glutamate uptake[57,81]. GRM1 was closely related to the RE pathological mechanism, which made it a potential new target for antiepileptic medications. The nicotine addiction pathway was also closely tied to the GABAergic synapse [82]. More than half of GABA receptors were distributed in module M155. Therefore, module M155 may provide the potential molecular network mechanism for nicotine-induced seizures.

Furthermore, the modules: M37, M80, M114, and M197, which had no available AEDs targets, also played central roles in the molecular network of RE. Functional analysis of these modules showed that M80 and M114 were mainly relevant to mTOR signaling pathway. This is consistent with that mTOR signaling pathway plays a role in RE physiopathology[83]; Module M37 was another significant network module because its most important function was oxidative phosphorylation—a function that was crucial for the development of epilepsy[84]; In our previous studies[23], four proteins (MT-CYB, UQCRB, UQCRC1 and UQCRH) were identified to be potential drug targets for RE. All these four proteins related to oxidative phosphorylation are the components of module M37. The genes of module M197 were mainly enriched in lipoprotein metabolism and HDL-mediated lipid transport pathways. A previous study showed that long-term AEDs therapy could significantly increase the total cholesterol and high-density lipoprotein cholesterol (HDLC). Moreover, the effect was more pronounced with HDLC[85]. Therefore, the further exploration of possible targets that interact with these modules would be much valuable for RE related drug pharmacological research.

However, our results would also be influenced by incomplete data and may contain bias introduced by available publications. As for the topology of modules, different algorithms may produce different community detection results that might influence some specific results. In addition, this study did not experimentally validate the 10 potential drug targets in vivo or vitro. This study is also not currently involved in clinical practice, but similar bioinformatics analysis has already applied to clinical cases [86]. However, we believe that our results provide novel insights on molecular mechanisms of RE. Furthermore, the curated RE-related genes and identified modules serve a useful resource for the discovery of potential drug targets for RE.

In the further research, we will validate some potential effective targets through molecular biology and animal model. We will also apply computer aided drug design (CADD) related software, such as DOCK, Auto Dock, and MOE, to facilitate potential drug design through docking computation[87]. It is well known that both RE and cancer have the issue of drug resistance in clinical practice. Previous studies showed that there were possible similar molecular mechanisms underlying the drug resistance in both RE and cancer [88]. We may reveal the potential molecular mechanism of drug resistance in RE and make comparison between RE and cancer through network analysis.

## Supporting information

S1 Table GO enrichment analysis- Biological Processes (BP) terms of RE seed genes. Here we just showed the top 200 BP terms (total 1014 BP terms).
(DOCX)

S2 Table GO enrichment analysis- Cellular Components (CC) terms of RE seed genes. Here we listed all the 214 CC terms.
(DOCX)

S3 Table GO enrichment analysis- Molecular Functions (MF) terms of RE seed genes. Here we listed all the 176 MF terms.
(DOCX)

S4 Table Reactome Pathway enrichment analysis of RE genes. (DOCX)

S5 Table Correlation between 185 modules and RE disorders. (DOCX)

S6 Table Primary 42 modules filtered by RR values. (DOCX)

S7 Table GO enrichment analysis of all 42 modules. We listed the smallest p-value terms of each module. If the smallest p-value >0.05, we leave it blank.
(DOCX)

S8 Table Combined score of module pair. (DOCX)

S9 Table Jaccard similarity between MeSH headings. Here we listed all the 193 records.
(DOCX)

S10 Table Drugs and targets distributed in modules. (DOCX)

## References

[1] Moshé SL, Perucca E, Ryvlin P, Tomson T. Epilepsy: new advances. Lancet.2015; 385 (14): 884–898. 25260236 10.1016/S0140-6736(14)60456-6

[2] Musto AE, Walker CP, Petasis NA, Bazan NG. Hippocampal neuro-networks and dendritic spine perturbations in epileptogenesis are attenuated by neuroprotectin d1. Plos One.2015; 10 (1): e0116543 10.1371/journal.pone.0116543 25617763 PMC4305283

[3] Bhalla D, Godet B, Druet-Cabanac M, Preux PM. Etiologies of epilepsy: a comprehensive review. Expert Review of Neurotherapeutics.2011; 11 (6): 861–876. 10.1586/ern.11.51 21651333

[4] Lim JS, Kim WI, Kang HC, Kim SH, Park AH, Park EK, et al Brain somatic mutations in MTOR cause focal cortical dysplasia type II leading to intractable epilepsy. Nature Medicine.2015; 21 (4): 395–400. 10.1038/nm.3824 25799227

[5] Peng YF, Song Z. NMDA receptor in the pathogenesis of epilepsy. Chinese Journal of Pathophysiology.2011; 27 (6): 1230–1229.

[6] Liu TT, He ZG, Tian XB, Xiang HB. Neural mechanisms and potential treatment of epilepsy and its complications. Am J Transl Res.2014; 6 (6): 625–630. 25628775 PMC4297332

[7] Quek AM, Britton JW, McKeon A, So E, Lennon VA, Shin C, et al Autoimmune epilepsy: clinical characteristics and response to immunotherapy. Arch Neurol.2012; 69 (5): 582–593. 10.1001/archneurol.2011.2985 22451162 PMC3601373

[8] Najjar S, Pearlman D, Miller DC, Devinsky O. Refractory epilepsy associated with microglial activation. Neurologist.2011; 17 (5): 249–254. 10.1097/NRL.0b013e31822aad04 21881466

[9] Ladino LD, Hernandez-Ronquillo L, Tellez-Zenteno JF. Management of antiepileptic drugs following epilepsy surgery: a meta-analysis. Epilepsy Res.2014; 108 (4): 765–774. 10.1016/j.eplepsyres.2014.01.024 24613746

[10] Ghaffarpour M, Ghelichnia HA, Harrichian MH, Ghabaee M, Tehrani MMS, Bahrami P. Strategies of Starting and Stopping Antiepileptic Drugs in Patients With Seizure or Epilepsy; a Comprehensive Review. Archives of Neuroscience.2014; 2 (1).

[11] Houman K, Christopher B, Parker DB, Snutch TP, Mcrory JE, Zamponi GW. Effects of Ca v 3.2 channel mutations linked to idiopathic generalized epilepsy. Ann Neurol.2005; 57 (5): 745–749. 10.1002/ana.20458 15852375

[12] Nelson M, Ming Y, Dowle AA, Thomas JR, Brackenbury WJ. The sodium channel-blocking antiepileptic drug phenytoin inhibits breast tumour growth and metastasis. Molecular Cancer.2015; 14 (1): 13–13. 25623198 10.1186/s12943-014-0277-x PMC4320839

[13] Qing-Ping W, Firas J, Agnès D, Jie G, Manuel S, Elisabeth D, et al Treatment of epilepsy: the GABA-transaminase inhibitor, vigabatrin, induces neuronal plasticity in the mouse retina. European Journal of Neuroscience.2008; 27 (8): 2177–2187. 10.1111/j.1460-9568.2008.06175.x 18412635 PMC2933832

[14] Chang HR, Kuo CC. Molecular Determinants of the Anticonvulsant Felbamate Binding Site in the N-Methyl-d-Aspartate Receptor. J Med Chem.2008; 51 (6): 1534–1545. 10.1021/jm0706618 18311896

[15] Rogawski MA, Loscher W. The neurobiology of antiepileptic drugs for the treatment of nonepileptic conditions. Nature Medicine.2004; 10 (7): 685–692. 10.1038/nm1074 15229516

[16] Merel W, Frans S S L, Toine C G E, Karel G M M, Sabine G U. Prognostic factors for medically intractable epilepsy: A systematic review. Epilepsy Research.2013; 106 (3): 301–310. 10.1016/j.eplepsyres.2013.06.013 23880113

[17] Beck H, Yaari Y Antiepileptogenesis, Plasticity of AED Targets, Drug Resistance, and Targeting the Immature Brain. 2012. 22787600

[18] Leu C, de Kovel CG, Zara F, Striano P, Pezzella M, Robbiano A, et al Genome-wide linkage meta-analysis identifies susceptibility loci at 2q34 and 13q31.3 for genetic generalized epilepsies. Epilepsia.2012; 53 (2): 308–318. 10.1111/j.1528-1167.2011.03379.x 22242659

[19] Anney R, Avbersek A, Balding D, Baum L, Becker F, Berkovic S, et al Genetic determinants of common epilepsies: a meta-analysis of genome-wide association studies: The Lancet Neurology. Lancet Neurology.2014; 13 (9): 893–903. 10.1016/S1474-4422(14)70171-1 25087078 PMC4189926

[20] Barabasi AL, Gulbahce N, Loscalzo J. Network medicine: a network-based approach to human disease. Nature Reviews Genetics.2011; 12 (1): 56–68. 10.1038/nrg2918 21164525 PMC3140052

[21] Sharma A, Gulbahce N, Pevzner SJ, Menche J, Ladenvall C, Folkersen L, et al Network based analysis of genome wide association data provides novel candidate genes for lipid and lipoprotein traits. Molecular & Cellular Proteomics Mcp.2013; 12 (11): 3398–3408. 23882023 10.1074/mcp.M112.024851 PMC3820950

[22] Menche J, Sharma A, Kitsak M, Ghiassian SD, Vidal M, Loscalzo J, et al Disease networks. Uncovering disease-disease relationships through the incomplete interactome. Science.2015; 347 (6224): 1257601 10.1126/science.1257601 25700523 PMC4435741

[23] Chu H, Zhou X, Liu G, Lv M, Zhou X, Wang Y, et al. Network-based detection of disease modules and potential drug targets in intractable epilepsy; 2014. pp. 132–140.

[24] Delahaye-Duriez A, Srivastava P, Shkura K, Langley SR, Laaniste L, Moreno-Moral A, et al Rare and common epilepsies converge on a shared gene regulatory network providing opportunities for novel antiepileptic drug discovery. Genome Biol.2016; 17 (1): 245 10.1186/s13059-016-1097-7 27955713 PMC5154105

[25] Lowe HJ, Barnett GO. Understanding and Using the Medical Subject Headings (MeSH) Vocabulary to Perform Literature Searches. Jama the Journal of the American Medical Association.1994; 271 (14): 1103–1108. 8151853

[26] Zhou X, Menche J, Barabasi AL, Sharma A. Human symptoms-disease network. Nature Communications.2014; 5: 4212 10.1038/ncomms5212 24967666

[27] Lu Z. PubMed and beyond: a survey of web tools for searching biomedical literature. Database (Oxford).2011; 2011: baq036. 21245076 10.1093/database/baq036 PMC3025693

[28] Hamosh A, Scott AF, Amberger JS, Bocchini CA, McKusick VA. Online Mendelian Inheritance in Man (OMIM), a knowledgebase of human genes and genetic disorders. Nucleic Acids Res.2005; 33 (Database issue): D514–517. 10.1093/nar/gki033 15608251 PMC539987

[29] Liu CC, Tseng YT, Li W, Wu CY, Mayzus I, Rzhetsky A, et al DiseaseConnect: a comprehensive web server for mechanism-based disease-disease connections. Nucleic Acids Res.2014; 42 (W1): W137–146. 24895436 10.1093/nar/gku412 PMC4086092

[30] Povey S, Lovering R, Bruford E, Wright M, Lush M, Wain H. The HUGO Gene Nomenclature Committee (HGNC). Human Genetics.2001; 109 (6): 678–680. 10.1007/s00439-001-0615-0 11810281

[31] Franceschini A, Szklarczyk D, Frankild S, Kuhn M, Simonovic M, Roth A, et al STRING v9.1: protein-protein interaction networks, with increased coverage and integration. Nucleic Acids Res.2013; 41 (Database issue): D808–815. 10.1093/nar/gks1094 23203871 PMC3531103

[32] Szklarczyk D, Franceschini A, Wyder S, Forslund K, Heller D, Huerta-Cepas J, et al STRING v10: protein-protein interaction networks, integrated over the tree of life. Nucleic Acids Res.2015; 43 (Database issue): D447–452. 10.1093/nar/gku1003 25352553 PMC4383874

[33] Von MC, Jensen LJ, Snel B, Hooper SD, Krupp M, Foglierini M, et al STRING: known and predicted protein—protein associations, integrated and transferred across organisms. Nucleic Acids Res.2005; 33 (suppl_1): 433–437. 10.1093/nar/gki005 PMC539959 15608232

[34] He D, Liu ZP, Chen L. Identification of dysfunctional modules and disease genes in congenital heart disease by a network-based approach. Bmc Genomics.2011; 12 (6): 813–819. 10.1186/1471-2164-12-592 PMC3256240 22136190

[35] Blondel VD, Guillaume JL, Lambiotte R, Lefebvre E. Fast unfolding of communities in large networks. Journal of Statistical Mechanics Theory & Experiment.2008; 30 (2): 155–168.

[36] Ashburner M, Ball CA, Blake JA, Botstein D, Butler H, Cherry JM, et al Gene ontology: tool for the unification of biology. The Gene Ontology Consortium. Nature Genetics.2000; 25 (1): 25–29. 10.1038/75556 10802651 PMC3037419

[37] Al-Shahrour F, Diaz-Uriarte R, Dopazo J. FatiGO: a web tool for finding significant associations of Gene Ontology terms with groups of genes. Bioinformatics.2004; 20 (4): 578–580. 10.1093/bioinformatics/btg455 14990455

[38] Shannon P, Markiel A, Ozier O, Baliga NS, Wang JT, Ramage D, et al Cytoscape: A Software Environment for Integrated Models of Biomolecular Interaction Networks. Genome Res.2003; 13 (11): 2498–2504. 10.1101/gr.1239303 14597658 PMC403769

[39] Maere S, Heymans K, Kuiper M. BiNGO: a Cytoscape plugin to assess overrepresentation of gene ontology categories in biological networks. Bioinformatics.2005; 21 (16): 3448–3449. 10.1093/bioinformatics/bti551 15972284

[40] Xie C, Mao X, Huang J, Ding Y, Wu J, Dong S, et al KOBAS 2.0: a web server for annotation and identification of enriched pathways and diseases. Nucleic Acids Res.2011; 39 (Web Server issue): W316–322. 10.1093/nar/gkr483 21715386 PMC3125809

[41] Joshi-Tope G, Gillespie M, Vastrik I, D'Eustachio P, Schmidt E, De BB, et al Reactome: a knowledgebase of biological pathways. Nucleic Acids Res.2005; 33 (Databaseissue): 428–432. 10.1093/nar/gki072 PMC540026 15608231

[42] Jacomy M, Venturini T, Heymann S, Bastian M. ForceAtlas2, a Continuous Graph Layout Algorithm for Handy Network Visualization Designed for the Gephi Software. Plos One.2014; 9 (6): e98679 10.1371/journal.pone.0098679 24914678 PMC4051631

[43] Law V, Knox C, Djoumbou Y, Jewison T, Guo AC, Liu YF, et al DrugBank 4.0: shedding new light on drug metabolism. Nucleic Acids Res.2014; 42 (D1): D1091–D1097. 24203711 10.1093/nar/gkt1068 PMC3965102

[44] Yildirim MA, Goh KI, Cusick ME, Barabasi AL, Vidal M. Drug-target network. Nature Biotechnology.2007; 25 (10): 1119–1126. 10.1038/nbt1338 17921997

[45] Girvan M, Newman ME. Community structure in social and biological networks. Proc Natl Acad Sci U S A.2002; 99 (12): 7821–7826. 10.1073/pnas.122653799 12060727 PMC122977

[46] DJ W, SH S. Collectivedynamics of ‘small-world’ networks; 1998. pp. 440–442.

[47] Cormen TH. Introduction to Algorithms. Mit Press 2001.

[48] Fisher RS, van Emde Boas W, Blume W, Elger C, Genton P, Lee P, et al Epileptic seizures and epilepsy: definitions proposed by the International League Against Epilepsy (ILAE) and the International Bureau for Epilepsy (IBE). Epilepsia.2005; 46 (4): 470–472. 10.1111/j.0013-9580.2005.66104.x 15816939

[49] Kerr M, Linehan C, Thompson R, Mula M, Gil-Nagal A, Zuberi SM, et al A White Paper on the medical and social needs of people with epilepsy and intellectual disability: the Task Force on Intellectual Disabilities and Epilepsy of the International League Against Epilepsy. Epilepsia.2014; 55 (12): 1902–1906. 10.1111/epi.12848 25378101

[50] Kohler S, Vasilevsky NA, Engelstad M, Foster E, McMurry J, Ayme S, et al The Human Phenotype Ontology in 2017. Nucleic Acids Res.2017; 45 (D1): D865–D876. 10.1093/nar/gkw1039 27899602 PMC5210535

[51] Guo L, Du Y, Wang J. Network analysis reveals a stress-affected common gene module among seven stress-related diseases/systems which provides potential targets for mechanism research. Scientific Reports.2015; 5: 12939 10.1038/srep12939 26245528 PMC4526881

[52] Porto JA, Oliveira AGD, Largura A, Adam TS, Nunes ML. Effects of epilepsy and malnutrition in the developing central nervous system: clinical aspects and experimental evidences. Journal of Epilepsy & Clinical Neurophysiology.2010; 16 (1): 26–31.

[53] Möhler H. GABAA receptors in central nervous system disease: anxiety, epilepsy, and insomnia. Journal of Receptors and Signal Transduction.2006; 26 (5–6): 731–740. 10.1080/10799890600920035 17118808

[54] Murphy JV, Patil AA. Improving the lives of patients with medically refractory epilepsy by electrical stimulation of the nervous system. Expert Review of Medical Devices.2014; 2 (2): 175–189. 10.1586/17434440.2.2.175 16293054

[55] Dingledine R, Varvel NH, Dudek FE. When and how do seizures kill neurons, and is cell death relevant to epileptogenesis? Adv Exp Med Biol.2014; 813: 109–122. 10.1007/978-94-017-8914-1_9 25012371 PMC4624106

[56] Nobili P. Seizure-induced pathologic plasticity and cell death in epileptogenic focal cortical dysplasia: converging evidences from human patients and an experimental model. Italy.2014.

[57] Rong L, Frontera AT, Benbadis SR. Tobacco smoking, epilepsy, and seizures. Epilepsy & Behavior.2014; 31 (2): 210–218. 24441294 10.1016/j.yebeh.2013.11.022

[58] Welch KA, Derry C. Mild traumatic brain injury and epilepsy: alcohol misuse may underpin the association. J Neurol Neurosurg Psychiatry.2014; 85 (6): 593 10.1136/jnnp-2013-306267 23892406

[59] Rogawski MA, Loscher W. The neurobiology of antiepileptic drugs. Nature Reviews Neuroscience.2004; 5 (7): 553–564. 10.1038/nrn1430 15208697

[60] Tan H, Orbak Z, Kantarci M, Koçak N, Karaca L. Valproate-induced insulin resistance in prepubertal girls with epilepsy. Journal of Pediatric Endocrinology and Metabolism.2014; 18 (10): 985–989. 10.1515/JPEM.2005.18.10.985 16355811

[61] Che F, Fu Q, Li X, Gao N, Qi F, Sun Z, et al Association of insulin receptor H1085H C>T, insulin receptor substrate 1 G972R and insulin receptor substrate 2 1057G/A polymorphisms with refractory temporal lobe epilepsy in Han Chinese. Seizure.2015; 25: 178–180. 10.1016/j.seizure.2014.09.014 25458098

[62] Steinlein OK. Nicotinic acetylcholine receptors and epilepsy. Current Drug Targets Cns & Neurological Disorders.2002; 1 (4): 443–448. 12769616 10.2174/1568007023339193

[63] Goh KI, Cusick ME, Valle D, Childs B, Vidal M, Barabási AL. The human disease network. Proceedings of the National Academy of Sciences of the United States of America.2007; 104 (21): 8685–8690. 10.1073/pnas.0701361104 17502601 PMC1885563

[64] Liu Y, Liu S. Protein-protein interaction network analysis of children atopic asthma. European Review for Medical & Pharmacological Sciences.2012; 16 (7): 867–872. 22953633

[65] Franceschini A, Szklarczyk D, Frankild S, Kuhn M, Simonovic M, Roth A, et al STRING v9.1: protein-protein interaction networks, with increased coverage and integration. Nucleic Acids Res.2013; 41 (Database issue): D808–815. 10.1093/nar/gks1094 23203871 PMC3531103

[66] Jeong H, Mason SP, Barabási AL, Oltvai ZN. Lethality and centrality in protein networks. Nature.2001; 411 (6833): 41–42. 10.1038/35075138 11333967

[67] Wang G, Bochorishvili G, Chen Y, Salvati KA, Zhang P, Dubel SJ, et al CaV3.2 calcium channels control NMDA receptor-mediated transmission: a new mechanism for absence epilepsy. Genes & Development.2015; 29 (14): 1535–1551. 26220996 10.1101/gad.260869.115 PMC4526737

[68] Gustafsson M, Nestor CE, Zhang H, Barabasi AL, Baranzini S, Brunak S, et al Modules, networks and systems medicine for understanding disease and aiding diagnosis. Genome Med.2014; 6: 82–82. 10.1186/s13073-014-0082-6 25473422 PMC4254417

[69] Sharma A, Menche J, Huang CC, Ort T, Zhou X, Kitsak M, et al A disease module in the interactome explains disease heterogeneity, drug response and captures novel pathways and genes in asthma. Human Molecular Genetics.2015; 24 (11): 3005–3020. 10.1093/hmg/ddv001 25586491 PMC4447811

[70] Oti M, Snel B, Huynen MA, Brunner HG. Predicting disease genes using protein-protein interactions. Journal of Medical Genetics.2006; 43 (8): 691–698. 10.1136/jmg.2006.041376 16611749 PMC2564594

[71] Myers CT, Mefford HC. Advancing epilepsy genetics in the genomic era. Genome Med.2015; 7: 91 10.1186/s13073-015-0214-7 26302787 PMC4549122

[72] Xu J, Li Y. Discovering disease-genes by topological features in human protein—protein interaction network. Bioinformatics.2006; 22 (22): 2800–2805. 10.1093/bioinformatics/btl467 16954137

[73] Jin SH, Jeong W, Chung CK. Mesial temporal lobe epilepsy with hippocampal sclerosis is a network disorder with altered cortical hubs. Epilepsia.2015; 56 (5): 772–779. 10.1111/epi.12966 25809843

[74] Guerrini R. Genetic Malformations of the Cerebral Cortex and Epilepsy. Epilepsia.2005; 46 suppl 1 (Supplement s1): 32–37. 15816977 10.1111/j.0013-9580.2005.461010.x

[75] Poucke MV, An EV, Peelman LJ, Ham LV. Experimental validation of in silico predicted KCNA1, KCNA2, KCNA6 and KCNQ2 genes for association studies of peripheral nerve hyperexcitability syndrome in Jack Russell Terriers. Neuromuscular Disorders.2012; 22 (6): 558–565. 10.1016/j.nmd.2012.01.008 22342001

[76] Abir T, Shigeki K, Maud B, Matthieu R, Katell F, Seishiro S, et al Cacnb4 directly couples electrical activity to gene expression, a process defective in juvenile epilepsy. Embo Journal.2012; 31 (18): 3730–3744. 10.1038/emboj.2012.226 22892567 PMC3442274

[77] Shu-Qiu W, Xiao-Jie L, Hong-Bin Q, Zhi-Mei J, Maria S, Xiao-Ru M, et al Anti-epileptic effect of Ganoderma lucidum polysaccharides by inhibition of intracellular calcium accumulation and stimulation of expression of CaMKII α in epileptic hippocampal neurons. Plos One.2014; 9 (7): e102161 10.1371/journal.pone.0102161 25010576 PMC4092074

[78] Dunn J, Blight A. Dalfampridine: a brief review of its mechanism of action and efficacy as a treatment to improve walking in patients with multiple sclerosis. Current Medical Research and Opinion.2011; 27 (7): 1415–1423. 10.1185/03007995.2011.583229 21595605

[79] Mancano MA. ISMP Adverse Drug Reactions—Dalfampridine-Induced Seizures; Efavirenz-Induced Hypersomnolence Leading to Coma and Death; Cerebral Toxoplasmosis with Rituximab; Torsades de Pointes after Low-Dose Aripiprazole; Atypical Antipsychotic-Induced Sleepwalking. Hospital Pharmacy.2013; 48 (9): 725–728.24421544

[80] And CFDE. Drug Safety and Availability—FDA Drug Safety Communication: Seizure risk for multiple sclerosis patients who take Ampyra (dalfampridine).

[81] Yoon HJ, Lim YJ, Zuo Z, Hur W, Do SH. Nicotine decreases the activity of glutamate transporter type 3. Toxicology Letters.2013; 225 (1): 147–152. 10.1016/j.toxlet.2013.12.002 24355585

[82] Ogata H, Goto S, Fujibuchi W, Kanehisa M. Computation with the KEGG pathway database. Biosystems.1998; 47 (1–2): 119–128. 9715755 10.1016/s0303-2647(98)00017-3

[83] Ostendorf AP, Wong M. mTOR Inhibition in Epilepsy: Rationale and Clinical Perspectives. Cns Drugs.2015; 29 (2): 91–99. 10.1007/s40263-014-0223-x 25633849 PMC4351152

[84] James J F, Michael J G, C Harker R, José M-G. Mitochondrial dysfunction in patients with hypotonia, epilepsy, autism, and developmental delay: HEADD syndrome. Journal of Child Neurology.2002; 17 (6): 435–439. 10.1177/088307380201700607 12174964

[85] Duggal HS, Jain R, Nizamie SH. Increased high-density lipoprotein cholesterol in patients with epilepsy treated with carbamazepine: a gender-related study. Epilepsia.1999; 40 (4): 480–484. 10219275 10.1111/j.1528-1157.1999.tb00744.x

[86] Striano P, Coppola A, Paravidino R, Malacarne M, Gimelli S, Robbiano A, et al Clinical significance of rare copy number variations in epilepsy: a case-control survey using microarray-based comparative genomic hybridization. Archives of Neurology.2012; 69 (3): 322–330. 10.1001/archneurol.2011.1999 22083797

[87] Hung CL, Chen CC. Computational Approaches for Drug Discovery. Drug Development Research.2014; 75 (6): 412–418. 10.1002/ddr.21222 25195585

[88] Sisodiya SM, Lin WR, Harding BN, Squier MV, Thom M. Drug resistance in epilepsy: expression of drug resistance proteins in common causes of refractory epilepsy. Brain.2002; 125 (Pt 1): 22–31. 11834590 10.1093/brain/awf002

