# Supplementary material for: Integrated network analysis reveals potentially novel molecular mechanisms and therapeutic targets of refractory epilepsies
Source: PLoS One. 2017 Apr 7;12(4):e0174964. doi: 10.1371/journal.pone.0174964 (PMC5384674; doi:10.1371/journal.pone.0174964)
Supplement: S1 Table — Here we just showed the top 200 BP terms (total 1014 BP terms). (DOCX) [file pone.0174964.s001.docx]

# S1 Table. GO enrichment analysis- biological processes (BP) terms of RE seed genes. Here we just showed the top 200 BP terms (total 1014 BP terms).

| **GO-ID** | **Description** | **p-value** |
| --- | --- | --- |
| 19226 | transmission of nerve impulse | 5.87E-52 |
| 7268 | synaptic transmission | 1.68E-46 |
| 7399 | nervous system development | 7.06E-41 |
| 48856 | anatomical structure development | 8.07E-35 |
| 48731 | system development | 1.59E-33 |
| 32501 | multicellular organismal process | 1.69E-33 |
| 7154 | cell communication | 2.00E-32 |
| 7267 | cell-cell signaling | 1.01E-30 |
| 32502 | developmental process | 2.49E-29 |
| 7275 | multicellular organismal development | 6.06E-29 |
| 23060 | signal transmission | 1.70E-28 |
| 23046 | signaling process | 1.70E-28 |
| 51179 | localization | 3.77E-28 |
| 22008 | neurogenesis | 2.73E-27 |
| 6811 | ion transport | 2.98E-27 |
| 48699 | generation of neurons | 4.40E-26 |
| 42391 | regulation of membrane potential | 3.18E-25 |
| 9987 | cellular process | 2.55E-24 |
| 51234 | establishment of localization | 1.16E-23 |
| 23052 | signaling | 1.40E-23 |
| 6810 | transport | 1.44E-23 |
| 7610 | behavior | 5.73E-23 |
| 65008 | regulation of biological quality | 7.57E-23 |
| 16043 | cellular component organization | 1.71E-22 |
| 30182 | neuron differentiation | 3.37E-22 |
| 48468 | cell development | 5.77E-21 |
| 7417 | central nervous system development | 2.19E-20 |
| 31644 | regulation of neurological system process | 4.53E-20 |
| 6873 | cellular ion homeostasis | 4.66E-20 |
| 50877 | neurological system process | 1.05E-19 |
| 55082 | cellular chemical homeostasis | 1.24E-19 |
| 3008 | system process | 4.95E-19 |
| 51969 | regulation of transmission of nerve impulse | 6.47E-19 |
| 45333 | cellular respiration | 1.68E-18 |
| 48869 | cellular developmental process | 1.75E-18 |
| 50801 | ion homeostasis | 3.57E-18 |
| 48666 | neuron development | 8.69E-18 |
| 9653 | anatomical structure morphogenesis | 8.69E-18 |
| 50804 | regulation of synaptic transmission | 9.15E-18 |
| 48878 | chemical homeostasis | 2.38E-17 |
| 19725 | cellular homeostasis | 2.86E-17 |
| 42592 | homeostatic process | 4.12E-17 |
| 44057 | regulation of system process | 4.32E-17 |
| 30154 | cell differentiation | 4.76E-17 |
| 48513 | organ development | 8.87E-17 |
| 7611 | learning or memory | 9.43E-17 |
| 65007 | biological regulation | 4.76E-16 |
| 30534 | adult behavior | 5.57E-16 |
| 6812 | cation transport | 8.50E-16 |
| 51239 | regulation of multicellular organismal process | 1.23E-15 |
| 904 | cell morphogenesis involved in differentiation | 4.09E-15 |
| 30001 | metal ion transport | 4.89E-15 |
| 42221 | response to chemical stimulus | 6.17E-15 |
| 30030 | cell projection organization | 8.05E-15 |
| 7420 | brain development | 9.61E-15 |
| 902 | cell morphogenesis | 1.19E-14 |
| 22607 | cellular component assembly | 1.25E-14 |
| 50767 | regulation of neurogenesis | 1.51E-14 |
| 22904 | respiratory electron transport chain | 1.64E-14 |
| 32989 | cellular component morphogenesis | 1.73E-14 |
| 60284 | regulation of cell development | 1.87E-14 |
| 15980 | energy derivation by oxidation of organic compounds | 2.19E-14 |
| 31175 | neuron projection development | 3.21E-14 |
| 45664 | regulation of neuron differentiation | 5.20E-14 |
| 51960 | regulation of nervous system development | 9.94E-14 |
| 6091 | generation of precursor metabolites and energy | 1.13E-13 |
| 10646 | regulation of cell communication | 1.61E-13 |
| 1508 | regulation of action potential | 1.62E-13 |
| 48667 | cell morphogenesis involved in neuron differentiation | 1.80E-13 |
| 48812 | neuron projection morphogenesis | 1.87E-13 |
| 32990 | cell part morphogenesis | 5.38E-13 |
| 48858 | cell projection morphogenesis | 1.18E-12 |
| 7612 | learning | 2.99E-12 |
| 7409 | axonogenesis | 5.38E-12 |
| 22900 | electron transport chain | 1.32E-11 |
| 16265 | death | 1.40E-11 |
| 21543 | pallium development | 2.07E-11 |
| 70271 | protein complex biogenesis | 2.07E-11 |
| 6461 | protein complex assembly | 2.07E-11 |
| 42981 | regulation of apoptosis | 2.13E-11 |
| 8219 | cell death | 2.19E-11 |
| 10941 | regulation of cell death | 2.73E-11 |
| 43067 | regulation of programmed cell death | 3.76E-11 |
| 44085 | cellular component biogenesis | 3.78E-11 |
| 48518 | positive regulation of biological process | 3.86E-11 |
| 48519 | negative regulation of biological process | 3.97E-11 |
| 42773 | ATP synthesis coupled electron transport | 4.03E-11 |
| 42775 | mitochondrial ATP synthesis coupled electron transport | 4.03E-11 |
| 30900 | forebrain development | 9.40E-11 |
| 8306 | associative learning | 9.53E-11 |
| 48522 | positive regulation of cellular process | 1.11E-10 |
| 10033 | response to organic substance | 1.24E-10 |
| 6836 | neurotransmitter transport | 1.27E-10 |
| 43523 | regulation of neuron apoptosis | 1.48E-10 |
| 50789 | regulation of biological process | 2.48E-10 |
| 8344 | adult locomotory behavior | 2.87E-10 |
| 65003 | macromolecular complex assembly | 2.91E-10 |
| 7214 | gamma-aminobutyric acid signaling pathway | 3.85E-10 |
| 1505 | regulation of neurotransmitter levels | 3.87E-10 |
| 7215 | glutamate signaling pathway | 4.87E-10 |
| 21537 | telencephalon development | 6.85E-10 |
| 51128 | regulation of cellular component organization | 1.05E-09 |
| 21987 | cerebral cortex development | 1.22E-09 |
| 50794 | regulation of cellular process | 1.80E-09 |
| 51049 | regulation of transport | 1.93E-09 |
| 6119 | oxidative phosphorylation | 2.48E-09 |
| 48523 | negative regulation of cellular process | 3.50E-09 |
| 65009 | regulation of molecular function | 3.54E-09 |
| 43933 | macromolecular complex subunit organization | 4.44E-09 |
| 60548 | negative regulation of cell death | 4.54E-09 |
| 43524 | negative regulation of neuron apoptosis | 5.28E-09 |
| 15807 | L-amino acid transport | 5.74E-09 |
| 32879 | regulation of localization | 5.74E-09 |
| 50790 | regulation of catalytic activity | 7.08E-09 |
| 50905 | neuromuscular process | 7.33E-09 |
| 35095 | behavioral response to nicotine | 7.33E-09 |
| 43092 | L-amino acid import | 7.33E-09 |
| 6120 | mitochondrial electron transport, NADH to ubiquinone | 7.72E-09 |
| 43279 | response to alkaloid | 1.03E-08 |
| 7270 | nerve-nerve synaptic transmission | 1.20E-08 |
| 51899 | membrane depolarization | 1.37E-08 |
| 7626 | locomotory behavior | 1.56E-08 |
| 6813 | potassium ion transport | 1.62E-08 |
| 48167 | regulation of synaptic plasticity | 2.00E-08 |
| 43066 | negative regulation of apoptosis | 2.42E-08 |
| 15672 | monovalent inorganic cation transport | 2.47E-08 |
| 60078 | regulation of postsynaptic membrane potential | 3.42E-08 |
| 6928 | cellular component movement | 3.57E-08 |
| 43069 | negative regulation of programmed cell death | 3.87E-08 |
| 43090 | amino acid import | 5.61E-08 |
| 7165 | signal transduction | 6.99E-08 |
| 44092 | negative regulation of molecular function | 6.99E-08 |
| 60341 | regulation of cellular localization | 6.99E-08 |
| 51896 | regulation of protein kinase B signaling cascade | 8.98E-08 |
| 45595 | regulation of cell differentiation | 9.16E-08 |
| 50896 | response to stimulus | 9.45E-08 |
| 51952 | regulation of amine transport | 1.22E-07 |
| 10975 | regulation of neuron projection development | 1.33E-07 |
| 7033 | vacuole organization | 1.39E-07 |
| 45471 | response to ethanol | 1.39E-07 |
| 35094 | response to nicotine | 1.39E-07 |
| 7005 | mitochondrion organization | 1.65E-07 |
| 9605 | response to external stimulus | 1.76E-07 |
| 70838 | divalent metal ion transport | 1.86E-07 |
| 14070 | response to organic cyclic substance | 2.05E-07 |
| 16310 | phosphorylation | 2.05E-07 |
| 1504 | neurotransmitter uptake | 2.35E-07 |
| 9790 | embryonic development | 2.75E-07 |
| 43623 | cellular protein complex assembly | 2.76E-07 |
| 9628 | response to abiotic stimulus | 2.88E-07 |
| 23033 | signaling pathway | 3.46E-07 |
| 6950 | response to stress | 3.86E-07 |
| 31344 | regulation of cell projection organization | 3.86E-07 |
| 6996 | organelle organization | 4.83E-07 |
| 15800 | acidic amino acid transport | 5.11E-07 |
| 51259 | protein oligomerization | 5.19E-07 |
| 60079 | regulation of excitatory postsynaptic membrane potential | 5.67E-07 |
| 21781 | glial cell fate commitment | 6.70E-07 |
| 6816 | calcium ion transport | 8.76E-07 |
| 6796 | phosphate metabolic process | 9.57E-07 |
| 6793 | phosphorus metabolic process | 9.57E-07 |
| 31667 | response to nutrient levels | 1.01E-06 |
| 50793 | regulation of developmental process | 1.06E-06 |
| 1764 | neuron migration | 1.08E-06 |
| 9991 | response to extracellular stimulus | 1.14E-06 |
| 51938 | L-glutamate import | 1.37E-06 |
| 19228 | regulation of action potential in neuron | 1.41E-06 |
| 16044 | cellular membrane organization | 1.60E-06 |
| 61024 | membrane organization | 1.73E-06 |
| 21761 | limbic system development | 1.77E-06 |
| 7628 | adult walking behavior | 1.96E-06 |
| 6084 | acetyl-CoA metabolic process | 2.12E-06 |
| 45665 | negative regulation of neuron differentiation | 2.12E-06 |
| 21953 | central nervous system neuron differentiation | 2.22E-06 |
| 21766 | hippocampus development | 2.24E-06 |
| 15674 | di-, tri-valent inorganic cation transport | 2.49E-06 |
| 51046 | regulation of secretion | 2.49E-06 |
| 51338 | regulation of transferase activity | 2.53E-06 |
| 43648 | dicarboxylic acid metabolic process | 2.57E-06 |
| 51186 | cofactor metabolic process | 2.71E-06 |
| 42493 | response to drug | 3.37E-06 |
| 23051 | regulation of signaling process | 4.33E-06 |
| 35249 | synaptic transmission, glutamatergic | 4.51E-06 |
| 14059 | regulation of dopamine secretion | 4.51E-06 |
| 15813 | L-glutamate transport | 4.51E-06 |
| 42220 | response to cocaine | 5.60E-06 |
| 14073 | response to tropane | 5.60E-06 |
| 30003 | cellular cation homeostasis | 6.78E-06 |
| 6105 | succinate metabolic process | 7.52E-06 |
| 9892 | negative regulation of metabolic process | 7.69E-06 |
| 61061 | muscle structure development | 7.99E-06 |
| 7271 | synaptic transmission, cholinergic | 7.99E-06 |
| 55080 | cation homeostasis | 8.99E-06 |
| 43085 | positive regulation of catalytic activity | 9.10E-06 |
| 10035 | response to inorganic substance | 1.12E-05 |
| 9966 | regulation of signal transduction | 1.18E-05 |
| 51641 | cellular localization | 1.19E-05 |
| 43086 | negative regulation of catalytic activity | 1.32E-05 |
| 35466 | regulation of signaling pathway | 1.38E-05 |
| 9887 | organ morphogenesis | 1.59E-05 |
