# Supplementary material for: Integrated network analysis reveals potentially novel molecular mechanisms and therapeutic targets of refractory epilepsies
Source: PLoS One. 2017 Apr 7;12(4):e0174964. doi: 10.1371/journal.pone.0174964 (PMC5384674; doi:10.1371/journal.pone.0174964)
Supplement: S2 Table — Here we listed all the 214 CC terms. (DOCX) [file pone.0174964.s002.docx]

# S2 Table. GO enrichment analysis- cellular components (CC) terms of RE seed genes. Here we listed all the 214 CC terms.

| **GO-ID** | **Description** | **p-value** |
| --- | --- | --- |
| 43005 | neuron projection | 2.91E-45 |
| 45202 | synapse | 4.64E-37 |
| 42995 | cell projection | 7.03E-35 |
| 5737 | cytoplasm | 5.00E-32 |
| 44456 | synapse part | 1.49E-31 |
| 44444 | cytoplasmic part | 2.08E-31 |
| 30425 | dendrite | 3.76E-29 |
| 44459 | plasma membrane part | 3.76E-29 |
| 34702 | ion channel complex | 2.17E-26 |
| 30424 | axon | 6.40E-26 |
| 44297 | cell body | 6.71E-24 |
| 43025 | neuronal cell body | 6.71E-24 |
| 43234 | protein complex | 1.29E-22 |
| 44463 | cell projection part | 5.99E-21 |
| 45211 | postsynaptic membrane | 5.99E-21 |
| 267 | cell fraction | 5.24E-19 |
| 32991 | macromolecular complex | 8.42E-18 |
| 5886 | plasma membrane | 1.02E-16 |
| 31226 | intrinsic to plasma membrane | 1.03E-16 |
| 5887 | integral to plasma membrane | 3.39E-16 |
| 30054 | cell junction | 3.65E-16 |
| 5626 | insoluble fraction | 4.97E-16 |
| 44429 | mitochondrial part | 4.02E-15 |
| 34703 | cation channel complex | 4.40E-15 |
| 5739 | mitochondrion | 1.17E-14 |
| 5624 | membrane fraction | 3.65E-14 |
| 5829 | cytosol | 1.21E-13 |
| 5740 | mitochondrial envelope | 3.00E-13 |
| 31975 | envelope | 3.70E-13 |
| 44424 | intracellular part | 3.70E-13 |
| 31966 | mitochondrial membrane | 6.47E-13 |
| 70469 | respiratory chain | 3.17E-12 |
| 19717 | synaptosome | 5.26E-12 |
| 31967 | organelle envelope | 1.62E-11 |
| 5746 | mitochondrial respiratory chain | 2.04E-11 |
| 5743 | mitochondrial inner membrane | 2.04E-11 |
| 31982 | vesicle | 2.55E-11 |
| 44455 | mitochondrial membrane part | 4.60E-11 |
| 19866 | organelle inner membrane | 5.61E-11 |
| 44422 | organelle part | 7.79E-11 |
| 31410 | cytoplasmic vesicle | 9.75E-11 |
| 5622 | intracellular | 1.06E-10 |
| 44446 | intracellular organelle part | 1.20E-10 |
| 43229 | intracellular organelle | 2.91E-10 |
| 33267 | axon part | 3.43E-10 |
| 43226 | organelle | 3.96E-10 |
| 14069 | postsynaptic density | 5.97E-10 |
| 44309 | neuron spine | 1.44E-09 |
| 45271 | respiratory chain complex I | 1.44E-09 |
| 5747 | mitochondrial respiratory chain complex I | 1.44E-09 |
| 43197 | dendritic spine | 1.44E-09 |
| 30964 | NADH dehydrogenase complex | 1.44E-09 |
| 42734 | presynaptic membrane | 1.45E-09 |
| 43231 | intracellular membrane-bounded organelle | 2.84E-09 |
| 43227 | membrane-bounded organelle | 3.29E-09 |
| 16023 | cytoplasmic membrane-bounded vesicle | 6.19E-09 |
| 43198 | dendritic shaft | 6.82E-09 |
| 31988 | membrane-bounded vesicle | 8.75E-09 |
| 5892 | nicotinic acetylcholine-gated receptor-channel complex | 1.43E-08 |
| 30666 | endocytic vesicle membrane | 1.61E-08 |
| 30426 | growth cone | 1.82E-08 |
| 30427 | site of polarized growth | 2.35E-08 |
| 8021 | synaptic vesicle | 3.12E-08 |
| 31090 | organelle membrane | 4.24E-08 |
| 5891 | voltage-gated calcium channel complex | 4.79E-08 |
| 45121 | membrane raft | 9.50E-08 |
| 30139 | endocytic vesicle | 1.14E-07 |
| 43235 | receptor complex | 1.74E-07 |
| 44433 | cytoplasmic vesicle part | 7.71E-07 |
| 43204 | perikaryon | 1.07E-06 |
| 5625 | soluble fraction | 1.28E-06 |
| 16020 | membrane | 1.35E-06 |
| 34704 | calcium channel complex | 1.47E-06 |
| 44430 | cytoskeletal part | 1.76E-06 |
| 8076 | voltage-gated potassium channel complex | 1.80E-06 |
| 34705 | potassium channel complex | 1.80E-06 |
| 323 | lytic vacuole | 2.04E-06 |
| 5764 | lysosome | 2.04E-06 |
| 5856 | cytoskeleton | 2.11E-06 |
| 31252 | cell leading edge | 2.12E-06 |
| 31974 | membrane-enclosed lumen | 2.52E-06 |
| 5773 | vacuole | 2.75E-06 |
| 30659 | cytoplasmic vesicle membrane | 3.17E-06 |
| 12506 | vesicle membrane | 3.45E-06 |
| 43195 | terminal button | 3.96E-06 |
| 9986 | cell surface | 6.43E-06 |
| 34707 | chloride channel complex | 7.44E-06 |
| 43233 | organelle lumen | 9.10E-06 |
| 8328 | ionotropic glutamate receptor complex | 1.26E-05 |
| 70013 | intracellular organelle lumen | 1.26E-05 |
| 44304 | main axon | 1.78E-05 |
| 30672 | synaptic vesicle membrane | 1.88E-05 |
| 5623 | cell | 2.14E-05 |
| 43679 | axon terminus | 2.49E-05 |
| 44306 | neuron projection terminus | 2.49E-05 |
| 42383 | sarcolemma | 2.59E-05 |
| 5759 | mitochondrial matrix | 3.11E-05 |
| 31980 | mitochondrial lumen | 3.11E-05 |
| 30136 | clathrin-coated vesicle | 3.14E-05 |
| 34706 | sodium channel complex | 3.82E-05 |
| 44464 | cell part | 3.82E-05 |
| 5901 | caveola | 4.56E-05 |
| 1518 | voltage-gated sodium channel complex | 1.09E-04 |
| 48471 | perinuclear region of cytoplasm | 1.32E-04 |
| 5938 | cell cortex | 1.93E-04 |
| 33268 | node of Ranvier | 2.44E-04 |
| 30135 | coated vesicle | 2.58E-04 |
| 43292 | contractile fiber | 3.32E-04 |
| 30665 | clathrin coated vesicle membrane | 3.71E-04 |
| 44224 | juxtaparanode region of axon | 6.74E-04 |
| 34399 | nuclear periphery | 7.09E-04 |
| 70369 | beta-catenin-TCF7L2 complex | 8.77E-04 |
| 15630 | microtubule cytoskeleton | 8.77E-04 |
| 32279 | asymmetric synapse | 9.06E-04 |
| 16363 | nuclear matrix | 9.88E-04 |
| 9897 | external side of plasma membrane | 1.01E-03 |
| 16010 | dystrophin-associated glycoprotein complex | 1.01E-03 |
| 44449 | contractile fiber part | 1.19E-03 |
| 30016 | myofibril | 1.32E-03 |
| 43034 | costamere | 1.41E-03 |
| 16234 | inclusion body | 1.93E-03 |
| 30315 | T-tubule | 1.93E-03 |
| 30175 | filopodium | 1.97E-03 |
| 30313 | cell envelope | 2.09E-03 |
| 44462 | external encapsulating structure part | 2.09E-03 |
| 30027 | lamellipodium | 2.09E-03 |
| 48770 | pigment granule | 2.21E-03 |
| 42470 | melanosome | 2.21E-03 |
| 16235 | aggresome | 2.43E-03 |
| 17146 | N-methyl-D-aspartate selective glutamate receptor complex | 2.43E-03 |
| 118 | histone deacetylase complex | 2.72E-03 |
| 31674 | I band | 2.72E-03 |
| 71664 | catenin-TCF7L2 complex | 2.72E-03 |
| 45281 | succinate dehydrogenase complex | 2.72E-03 |
| 45283 | fumarate reductase complex | 2.72E-03 |
| 45257 | succinate dehydrogenase complex (ubiquinone) | 2.72E-03 |
| 45273 | respiratory chain complex II | 2.72E-03 |
| 5749 | mitochondrial respiratory chain complex II | 2.72E-03 |
| 43083 | synaptic cleft | 2.72E-03 |
| 44421 | extracellular region part | 2.74E-03 |
| 30018 | Z disc | 2.74E-03 |
| 30312 | external encapsulating structure | 2.74E-03 |
| 43228 | non-membrane-bounded organelle | 2.90E-03 |
| 43232 | intracellular non-membrane-bounded organelle | 2.90E-03 |
| 30864 | cortical actin cytoskeleton | 2.96E-03 |
| 32839 | dendrite cytoplasm | 3.73E-03 |
| 32589 | neuron projection membrane | 3.80E-03 |
| 31256 | leading edge membrane | 4.98E-03 |
| 5881 | cytoplasmic microtubule | 5.21E-03 |
| 5765 | lysosomal membrane | 5.42E-03 |
| 42597 | periplasmic space | 5.68E-03 |
| 32838 | cell projection cytoplasm | 5.68E-03 |
| 30288 | outer membrane-bounded periplasmic space | 5.68E-03 |
| 30662 | coated vesicle membrane | 5.74E-03 |
| 30017 | sarcomere | 5.78E-03 |
| 31588 | AMP-activated protein kinase complex | 5.78E-03 |
| 31512 | motile primary cilium | 5.78E-03 |
| 44425 | membrane part | 6.26E-03 |
| 5758 | mitochondrial intermembrane space | 8.08E-03 |
| 5768 | endosome | 8.28E-03 |
| 1726 | ruffle | 8.37E-03 |
| 31232 | extrinsic to external side of plasma membrane | 1.05E-02 |
| 2133 | polycystin complex | 1.05E-02 |
| 32281 | alpha-amino-3-hydroxy-5-methyl-4-isoxazolepropionic acid selective glutamate receptor complex | 1.05E-02 |
| 32809 | neuronal cell body membrane | 1.05E-02 |
| 33596 | TSC1-TSC2 complex | 1.05E-02 |
| 5899 | insulin receptor complex | 1.05E-02 |
| 44298 | cell body membrane | 1.05E-02 |
| 48237 | rough endoplasmic reticulum lumen | 1.05E-02 |
| 31932 | TORC2 complex | 1.05E-02 |
| 1940 | male pronucleus | 1.05E-02 |
| 44420 | extracellular matrix part | 1.05E-02 |
| 5654 | nucleoplasm | 1.12E-02 |
| 30863 | cortical cytoskeleton | 1.22E-02 |
| 44437 | vacuolar part | 1.31E-02 |
| 5615 | extracellular space | 1.59E-02 |
| 15629 | actin cytoskeleton | 1.60E-02 |
| 16011 | dystroglycan complex | 1.64E-02 |
| 5874 | microtubule | 1.66E-02 |
| 31970 | organelle envelope lumen | 1.86E-02 |
| 5774 | vacuolar membrane | 1.87E-02 |
| 31528 | microvillus membrane | 1.90E-02 |
| 307 | cyclin-dependent protein kinase holoenzyme complex | 1.90E-02 |
| 31225 | anchored to membrane | 2.06E-02 |
| 5667 | transcription factor complex | 2.16E-02 |
| 30141 | stored secretory granule | 2.19E-02 |
| 5813 | centrosome | 2.22E-02 |
| 31253 | cell projection membrane | 2.31E-02 |
| 35253 | ciliary rootlet | 2.34E-02 |
| 16580 | Sin3 complex | 2.34E-02 |
| 5652 | nuclear lamina | 2.34E-02 |
| 70822 | Sin3-type complex | 2.34E-02 |
| 5815 | microtubule organizing center | 2.44E-02 |
| 16533 | cyclin-dependent protein kinase 5 holoenzyme complex | 2.63E-02 |
| 31045 | dense core granule | 2.63E-02 |
| 45254 | pyruvate dehydrogenase complex | 2.63E-02 |
| 70852 | cell body fiber | 2.63E-02 |
| 1939 | female pronucleus | 2.63E-02 |
| 31594 | neuromuscular junction | 2.63E-02 |
| 16324 | apical plasma membrane | 2.63E-02 |
| 32994 | protein-lipid complex | 2.87E-02 |
| 34358 | plasma lipoprotein particle | 2.87E-02 |
| 19898 | extrinsic to membrane | 2.98E-02 |
| 31981 | nuclear lumen | 2.98E-02 |
| 45177 | apical part of cell | 3.11E-02 |
| 8091 | spectrin | 3.13E-02 |
| 5788 | endoplasmic reticulum lumen | 3.18E-02 |
| 5845 | mRNA cap binding complex | 4.22E-02 |
| 34518 | RNA cap binding complex | 4.22E-02 |
| 299 | integral to membrane of membrane fraction | 4.22E-02 |
| 5844 | polysome | 4.22E-02 |
| 32590 | dendrite membrane | 4.68E-02 |
| 19908 | nuclear cyclin-dependent protein kinase holoenzyme complex | 4.68E-02 |
| 5827 | polar microtubule | 4.68E-02 |
