# Supplementary material for: Integrated network analysis reveals potentially novel molecular mechanisms and therapeutic targets of refractory epilepsies
Source: PLoS One. 2017 Apr 7;12(4):e0174964. doi: 10.1371/journal.pone.0174964 (PMC5384674; doi:10.1371/journal.pone.0174964)
Supplement: S3 Table — Here we listed all the 176 MF terms. (DOCX) [file pone.0174964.s003.docx]

# S3 Table. GO enrichment analysis- molecular functions (MF) terms of RE seed genes. Here we listed all the 176 MF terms.

| **GO-ID** | **Description** | **p-value** |
| --- | --- | --- |
| 22836 | gated channel activity | 4.31E-35 |
| 5216 | ion channel activity | 2.36E-32 |
| 15267 | channel activity | 2.36E-32 |
| 22838 | substrate-specific channel activity | 2.36E-32 |
| 22803 | passive transmembrane transporter activity | 2.36E-32 |
| 15075 | ion transmembrane transporter activity | 7.48E-30 |
| 22891 | substrate-specific transmembrane transporter activity | 1.09E-27 |
| 5515 | protein binding | 1.09E-26 |
| 22857 | transmembrane transporter activity | 7.18E-26 |
| 22892 | substrate-specific transporter activity | 4.52E-25 |
| 5261 | cation channel activity | 2.36E-24 |
| 8324 | cation transmembrane transporter activity | 2.09E-23 |
| 5215 | transporter activity | 3.95E-23 |
| 22843 | voltage-gated cation channel activity | 3.64E-20 |
| 5230 | extracellular ligand-gated ion channel activity | 3.65E-20 |
| 22832 | voltage-gated channel activity | 4.77E-20 |
| 5244 | voltage-gated ion channel activity | 4.77E-20 |
| 15276 | ligand-gated ion channel activity | 9.93E-20 |
| 22834 | ligand-gated channel activity | 9.93E-20 |
| 8066 | glutamate receptor activity | 1.51E-17 |
| 5231 | excitatory extracellular ligand-gated ion channel activity | 9.97E-15 |
| 42165 | neurotransmitter binding | 2.35E-14 |
| 43176 | amine binding | 2.05E-10 |
| 19899 | enzyme binding | 7.73E-09 |
| 16247 | channel regulator activity | 8.46E-09 |
| 42166 | acetylcholine binding | 9.75E-09 |
| 30594 | neurotransmitter receptor activity | 9.75E-09 |
| 5267 | potassium channel activity | 1.04E-08 |
| 42802 | identical protein binding | 1.66E-08 |
| 46983 | protein dimerization activity | 1.92E-08 |
| 5272 | sodium channel activity | 2.65E-08 |
| 5234 | extracellular-glutamate-gated ion channel activity | 3.83E-08 |
| 8137 | NADH dehydrogenase (ubiquinone) activity | 3.83E-08 |
| 50136 | NADH dehydrogenase (quinone) activity | 3.83E-08 |
| 3954 | NADH dehydrogenase activity | 3.83E-08 |
| 19904 | protein domain specific binding | 5.26E-08 |
| 4889 | nicotinic acetylcholine-activated cation-selective channel activity | 6.98E-08 |
| 5488 | binding | 1.48E-07 |
| 4970 | ionotropic glutamate receptor activity | 1.52E-07 |
| 5249 | voltage-gated potassium channel activity | 1.58E-07 |
| 16917 | GABA receptor activity | 2.41E-07 |
| 16655 | oxidoreductase activity, acting on NADH or NADPH, quinone or similar compound as acceptor | 2.72E-07 |
| 5262 | calcium channel activity | 5.12E-07 |
| 5248 | voltage-gated sodium channel activity | 6.45E-07 |
| 5245 | voltage-gated calcium channel activity | 6.99E-07 |
| 15464 | acetylcholine receptor activity | 1.34E-06 |
| 51539 | 4 iron, 4 sulfur cluster binding | 1.86E-06 |
| 8509 | anion transmembrane transporter activity | 4.26E-06 |
| 16595 | glutamate binding | 5.67E-06 |
| 4890 | GABA-A receptor activity | 8.49E-06 |
| 16597 | amino acid binding | 1.16E-05 |
| 5254 | chloride channel activity | 2.47E-05 |
| 51536 | iron-sulfur cluster binding | 2.49E-05 |
| 51540 | metal cluster binding | 2.49E-05 |
| 42803 | protein homodimerization activity | 6.09E-05 |
| 8022 | protein C-terminus binding | 7.89E-05 |
| 16651 | oxidoreductase activity, acting on NADH or NADPH | 8.18E-05 |
| 5253 | anion channel activity | 8.81E-05 |
| 22890 | inorganic cation transmembrane transporter activity | 1.19E-04 |
| 15081 | sodium ion transmembrane transporter activity | 1.46E-04 |
| 43565 | sequence-specific DNA binding | 1.55E-04 |
| 8092 | cytoskeletal protein binding | 1.57E-04 |
| 19900 | kinase binding | 1.77E-04 |
| 5102 | receptor binding | 1.84E-04 |
| 31406 | carboxylic acid binding | 1.98E-04 |
| 15277 | kainate selective glutamate receptor activity | 2.10E-04 |
| 15459 | potassium channel regulator activity | 2.12E-04 |
| 5416 | cation:amino acid symporter activity | 2.21E-04 |
| 46873 | metal ion transmembrane transporter activity | 2.40E-04 |
| 5246 | calcium channel regulator activity | 2.46E-04 |
| 15077 | monovalent inorganic cation transmembrane transporter activity | 2.57E-04 |
| 46982 | protein heterodimerization activity | 2.93E-04 |
| 30165 | PDZ domain binding | 3.12E-04 |
| 15370 | solute:sodium symporter activity | 4.70E-04 |
| 15294 | solute:cation symporter activity | 5.72E-04 |
| 15185 | L-gamma-aminobutyric acid transmembrane transporter activity | 7.88E-04 |
| 1640 | adenylate cyclase inhibiting metabotropic glutamate receptor activity | 7.88E-04 |
| 15293 | symporter activity | 8.70E-04 |
| 5283 | sodium:amino acid symporter activity | 9.56E-04 |
| 5200 | structural constituent of cytoskeleton | 1.03E-03 |
| 5343 | organic acid:sodium symporter activity | 1.04E-03 |
| 19901 | protein kinase binding | 1.13E-03 |
| 5328 | neurotransmitter:sodium symporter activity | 1.15E-03 |
| 32403 | protein complex binding | 1.62E-03 |
| 15179 | L-amino acid transmembrane transporter activity | 1.66E-03 |
| 8144 | drug binding | 1.81E-03 |
| 8503 | benzodiazepine receptor activity | 2.00E-03 |
| 8200 | ion channel inhibitor activity | 2.30E-03 |
| 48039 | ubiquinone binding | 2.41E-03 |
| 4351 | glutamate decarboxylase activity | 2.41E-03 |
| 4971 | alpha-amino-3-hydroxy-5-methyl-4-isoxazole propionate selective glutamate receptor activity | 2.41E-03 |
| 8332 | low voltage-gated calcium channel activity | 2.41E-03 |
| 48038 | quinone binding | 2.67E-03 |
| 5313 | L-glutamate transmembrane transporter activity | 2.67E-03 |
| 16248 | channel inhibitor activity | 3.14E-03 |
| 48037 | cofactor binding | 3.55E-03 |
| 5326 | neurotransmitter transporter activity | 3.58E-03 |
| 31405 | lipoic acid binding | 3.95E-03 |
| 15172 | acidic amino acid transmembrane transporter activity | 4.15E-03 |
| 15296 | anion:cation symporter activity | 4.15E-03 |
| 15291 | secondary active transmembrane transporter activity | 4.43E-03 |
| 16301 | kinase activity | 4.96E-03 |
| 51219 | phosphoprotein binding | 5.05E-03 |
| 8134 | transcription factor binding | 6.94E-03 |
| 43121 | neurotrophin binding | 7.03E-03 |
| 16491 | oxidoreductase activity | 7.32E-03 |
| 104 | succinate dehydrogenase activity | 7.32E-03 |
| 30229 | very-low-density lipoprotein receptor activity | 7.32E-03 |
| 70326 | very-low-density lipoprotein receptor binding | 7.32E-03 |
| 42910 | xenobiotic transporter activity | 7.32E-03 |
| 34189 | very-low-density lipoprotein binding | 7.32E-03 |
| 5237 | inhibitory extracellular ligand-gated ion channel activity | 7.32E-03 |
| 5332 | gamma-aminobutyric acid:sodium symporter activity | 7.32E-03 |
| 1601 | peptide YY receptor activity | 7.32E-03 |
| 3696 | satellite DNA binding | 7.32E-03 |
| 15467 | G-protein activated inward rectifier potassium channel activity | 7.32E-03 |
| 19205 | nucleobase, nucleoside, nucleotide kinase activity | 7.59E-03 |
| 5159 | insulin-like growth factor receptor binding | 7.98E-03 |
| 5275 | amine transmembrane transporter activity | 8.57E-03 |
| 5242 | inward rectifier potassium channel activity | 8.93E-03 |
| 4672 | protein kinase activity | 1.06E-02 |
| 16773 | phosphotransferase activity, alcohol group as acceptor | 1.06E-02 |
| 8017 | microtubule binding | 1.36E-02 |
| 19201 | nucleotide kinase activity | 1.46E-02 |
| 15171 | amino acid transmembrane transporter activity | 1.48E-02 |
| 4697 | protein kinase C activity | 1.48E-02 |
| 15631 | tubulin binding | 1.53E-02 |
| 43274 | phospholipase binding | 1.53E-02 |
| 31420 | alkali metal ion binding | 1.53E-02 |
| 17153 | sodium:dicarboxylate symporter activity | 1.53E-02 |
| 4972 | N-methyl-D-aspartate selective glutamate receptor activity | 1.53E-02 |
| 5030 | neurotrophin receptor activity | 1.53E-02 |
| 31402 | sodium ion binding | 1.53E-02 |
| 5509 | calcium ion binding | 1.58E-02 |
| 46943 | carboxylic acid transmembrane transporter activity | 1.61E-02 |
| 5342 | organic acid transmembrane transporter activity | 1.77E-02 |
| 4385 | guanylate kinase activity | 2.19E-02 |
| 19894 | kinesin binding | 2.19E-02 |
| 15222 | serotonin transmembrane transporter activity | 2.48E-02 |
| 2151 | G-quadruplex RNA binding | 2.48E-02 |
| 19811 | cocaine binding | 2.48E-02 |
| 50347 | trans-octaprenyltranstransferase activity | 2.48E-02 |
| 400 | four-way junction DNA binding | 2.48E-02 |
| 19948 | SUMO activating enzyme activity | 2.48E-02 |
| 8240 | tripeptidyl-peptidase activity | 2.48E-02 |
| 8327 | methyl-CpG binding | 2.48E-02 |
| 42835 | BRE binding | 2.48E-02 |
| 16635 | oxidoreductase activity, acting on the CH-CH group of donors, quinone or related compound as acceptor | 2.48E-02 |
| 5314 | high-affinity glutamate transmembrane transporter activity | 2.48E-02 |
| 5335 | serotonin:sodium symporter activity | 2.48E-02 |
| 1642 | group III metabotropic glutamate receptor activity | 2.48E-02 |
| 1641 | group II metabotropic glutamate receptor activity | 2.48E-02 |
| 8177 | succinate dehydrogenase (ubiquinone) activity | 2.48E-02 |
| 46403 | polynucleotide 3'-phosphatase activity | 2.48E-02 |
| 3690 | double-stranded DNA binding | 2.48E-02 |
| 30544 | Hsp70 protein binding | 2.48E-02 |
| 51739 | ammonia transmembrane transporter activity | 2.48E-02 |
| 70412 | R-SMAD binding | 2.71E-02 |
| 43560 | insulin receptor substrate binding | 2.71E-02 |
| 48365 | Rac GTPase binding | 2.71E-02 |
| 3824 | catalytic activity | 2.79E-02 |
| 50839 | cell adhesion molecule binding | 2.83E-02 |
| 47485 | protein N-terminus binding | 3.16E-02 |
| 17124 | SH3 domain binding | 3.38E-02 |
| 3700 | transcription factor activity | 3.40E-02 |
| 16772 | transferase activity, transferring phosphorus-containing groups | 3.40E-02 |
| 22804 | active transmembrane transporter activity | 3.41E-02 |
| 50750 | low-density lipoprotein receptor binding | 3.56E-02 |
| 32947 | protein complex scaffold | 3.74E-02 |
| 51287 | NAD or NADH binding | 3.86E-02 |
| 46332 | SMAD binding | 3.86E-02 |
| 9055 | electron carrier activity | 4.06E-02 |
| 51087 | chaperone binding | 4.35E-02 |
| 43014 | alpha-tubulin binding | 4.60E-02 |
| 16566 | specific transcriptional repressor activity | 4.77E-02 |
| 8188 | neuropeptide receptor activity | 4.77E-02 |
