# Supplementary material for: Integrated network analysis reveals potentially novel molecular mechanisms and therapeutic targets of refractory epilepsies
Source: PLoS One. 2017 Apr 7;12(4):e0174964. doi: 10.1371/journal.pone.0174964 (PMC5384674; doi:10.1371/journal.pone.0174964)
Supplement: S4 Table — (DOCX) [file pone.0174964.s004.docx]

# S4 Table. Reactome Pathway enrichment analysis of RE genes.

| **Term** | **ID** | **P-Value** | **Corrected P-Value** |
| --- | --- | --- | --- |
| Neuronal System | REACT_13685 | 1.71E-25 | 6.27E-22 |
| Transmission across Chemical Synapses | REACT_13477 | 1.77E-22 | 3.52E-19 |
| Neurotransmitter Receptor Binding And Downstream Transmission In The Postsynaptic Cell | REACT_15370 | 2.09E-15 | 7.92E-13 |
| The citric acid (TCA) cycle and respiratory electron transport | REACT_111083 | 1.00E-09 | 1.10E-07 |
| L1CAM interactions | REACT_22205 | 1.08E-08 | 9.82E-07 |
| Respiratory electron transport | REACT_22393 | 3.25E-08 | 2.67E-06 |
| Respiratory electron transport, ATP synthesis by chemiosmotic coupling, and heat production by uncoupling proteins. | REACT_6305 | 1.69E-07 | 1.27E-05 |
| Axon guidance | REACT_18266 | 3.38E-07 | 2.43E-05 |
| mTOR signalling | REACT_6838 | 1.77E-06 | 0.0001066 |
| Highly calcium permeable postsynaptic nicotinic acetylcholine receptors | REACT_22303 | 2.05E-06 | 0.0001222 |
| PKB-mediated events | REACT_456 | 2.44E-06 | 0.000143 |
| Potassium Channels | REACT_75908 | 3.03E-06 | 0.0001727 |
| GABA A receptor activation | REACT_24987 | 3.38E-06 | 0.0001868 |
| @@GABA receptor activation | REACT_25199 | 2.70E-05 | 0.0010336 |
| Unblocking of NMDA receptor, glutamate binding and activation | REACT_20594 | 3.63E-06 | 0.0001978 |
| Interaction between L1 and Ankyrins | REACT_22266 | 4.49E-06 | 0.0002408 |
| Postsynaptic nicotinic acetylcholine receptors | REACT_22149 | 8.36E-06 | 0.0003916 |
| Activation of Nicotinic Acetylcholine Receptors | REACT_22126 | 8.36E-06 | 0.0003916 |
| Acetylcholine Binding And Downstream Events | REACT_15461 | 8.36E-06 | 0.0003916 |
| Neurotransmitter Release Cycle | REACT_13723 | 1.59E-05 | 0.000685 |
| Highly calcium permeable nicotinic acetylcholine receptors | REACT_22352 | 1.63E-05 | 0.0006971 |
| Insulin receptor signalling cascade | REACT_1195 | 1.75E-05 | 0.0007366 |
| Glutamate Binding, Activation of AMPA Receptors and Synaptic Plasticity | REACT_18347 | 1.81E-05 | 0.0007553 |
| Ligand-gated ion channel transport | REACT_25387 | 1.81E-05 | 0.0007553 |
| Trafficking of AMPA receptors | REACT_18307 | 1.81E-05 | 0.0007553 |
| Developmental Biology | REACT_111045 | 2.02E-05 | 0.0008149 |
| IRS-related events | REACT_762 | 2.61E-05 | 0.0010183 |
| PI3K Cascade | REACT_976 | 2.66E-05 | 0.001026 |
| GABA synthesis, release, reuptake and degradation | REACT_23947 | 2.70E-05 | 0.0010336 |
| Citric acid cycle (TCA cycle) | REACT_1785 | 3.84E-05 | 0.0013685 |
| Activation of NMDA receptor upon glutamate binding and postsynaptic events | REACT_20563 | 5.60E-05 | 0.0019266 |
| Depolarization of the Presynaptic Terminal Triggers the Opening of Calcium Channels | REACT_13606 | 6.85E-05 | 0.0022717 |
| Presynaptic nicotinic acetylcholine receptors | REACT_22336 | 6.85E-05 | 0.0022717 |
| Signaling by Insulin receptor | REACT_498 | 7.25E-05 | 0.0023845 |
| Pyruvate metabolism and Citric Acid (TCA) cycle | REACT_1046 | 8.55E-05 | 0.0027191 |
| Energy dependent regulation of mTOR by LKB1-AMPK | REACT_21387 | 9.09E-05 | 0.0028496 |
| IRS-mediated signalling | REACT_332 | 0.0001243 | 0.0037082 |
| Regulation of Rheb GTPase activity by AMPK | REACT_21393 | 0.0001596 | 0.0046593 |
| IRS-related events triggered by IGF1R | REACT_150203 | 0.0002175 | 0.005911 |
| Signaling by Type 1 Insulin-like Growth Factor 1 Receptor (IGF1R) | REACT_150359 | 0.000323 | 0.0080416 |
| IGF1R signaling cascade | REACT_150210 | 0.000323 | 0.0080416 |
| CREB phosphorylation through the activation of CaMKII | REACT_20642 | 0.0004144 | 0.0099583 |
| CREB phosphorylation through the activation of Ras | REACT_20568 | 0.0005964 | 0.0135334 |
| Signalling by NGF | REACT_11061 | 0.0007231 | 0.0157569 |
| Class C/3 (Metabotropic glutamate/pheromone receptors) | REACT_18319 | 0.0010824 | 0.0218162 |
| NrCAM interactions | REACT_22329 | 0.0013749 | 0.0267583 |
| Trafficking of GluR2-containing AMPA receptors | REACT_18422 | 0.0017511 | 0.03198 |
| mTORC1-mediated signalling | REACT_6964 | 0.0017511 | 0.03198 |
| Activation of Ca-permeable Kainate Receptor | REACT_21346 | 0.0017511 | 0.03198 |
| Ionotropic activity of Kainate Receptors | REACT_21322 | 0.0017511 | 0.03198 |
| NCAM signaling for neurite out-growth | REACT_18334 | 0.0023497 | 0.0402787 |
| CD28 co-stimulation | REACT_19183 | 0.0024628 | 0.0414417 |
| Voltage gated Potassium channels | REACT_75770 | 0.002488 | 0.0418016 |
| Post NMDA receptor activation events | REACT_20593 | 0.0027535 | 0.0450922 |
| Ras activation uopn Ca2+ infux through NMDA receptor | REACT_20546 | 0.0029087 | 0.0465944 |
