# Supplementary material for: Integrated network analysis reveals potentially novel molecular mechanisms and therapeutic targets of refractory epilepsies
Source: PLoS One. 2017 Apr 7;12(4):e0174964. doi: 10.1371/journal.pone.0174964 (PMC5384674; doi:10.1371/journal.pone.0174964)
Supplement: S5 Table — (DOCX) [file pone.0174964.s005.docx]

# S5 Table. Correlation between 185 modules and RE disorders.

| **Module** | **Number of proteins** | **Number of seed genes** | **Chi-Square** | **corrected p-value** |
| --- | --- | --- | --- | --- |
| 145 | 7 | 7 | 84.89282 | 9.06E-322 |
| 155 | 141 | 40 | 87.21958 | 9.06E-322 |
| 65 | 184 | 36 | 37.85565 | 1.40928E-07 |
| 188 | 43 | 12 | 25.22096 | 9.45787E-05 |
| 37 | 305 | 45 | 22.60791 | 0.00036753 |
| 197 | 50 | 11 | 14.74245 | 0.022799356 |
| 208 | 34 | 8 | 12.25467 | 0.085857505 |
| 80 | 52 | 10 | 9.99668 | 0.290122048 |
| 304 | 262 | 7 | 9.319519 | 0.41944214 |
| 306 | 214 | 5 | 8.643258 | 0.60730663 |
| 83 | 5 | 2 | 7.448007 | 1.174874397 |
| 42 | 158 | 3 | 7.44712 | 1.175453561 |
| 271 | 16 | 4 | 6.872163 | 1.619649831 |
| 31 | 149 | 3 | 6.73917 | 1.744892418 |
| 114 | 53 | 9 | 6.623225 | 1.862161908 |
| 192 | 97 | 1 | 6.032796 | 2.597857655 |
| 170 | 155 | 4 | 5.667 | 3.198093997 |
| 129 | 6 | 2 | 5.636492 | 3.254218243 |
| 150 | 6 | 2 | 5.636492 | 3.254218243 |
| 74 | 113 | 2 | 5.547652 | 3.423516887 |
| 55 | 131 | 17 | 5.394833 | 3.736343958 |
| 84 | 89 | 1 | 5.377097 | 3.774518882 |
| 104 | 123 | 16 | 5.120821 | 4.373479902 |
| 49 | 200 | 7 | 4.906828 | 4.948883565 |
| 27 | 19 | 4 | 4.875661 | 5.03903215 |
| 3 | 140 | 4 | 4.566732 | 6.030758924 |
| 183 | 139 | 4 | 4.494698 | 6.290020296 |
| 22 | 78 | 1 | 4.481374 | 6.339240247 |
| 59 | 77 | 1 | 4.400347 | 6.647349904 |
| 253 | 36 | 6 | 4.195638 | 7.497697862 |
| 168 | 152 | 5 | 4.103306 | 7.917898933 |
| 29 | 133 | 4 | 4.066479 | 8.092335204 |
| 230 | 45 | 7 | 4.038201 | 8.229025582 |
| 149 | 92 | 2 | 3.907687 | 8.89215366 |
| 101 | 167 | 6 | 3.903911 | 8.91216097 |
| 26 | 21 | 4 | 3.900693 | 8.92924624 |
| 223 | 55 | 8 | 3.761963 | 9.699731733 |
| 120 | 88 | 2 | 3.60222 | 10.67495787 |
| 36 | 66 | 1 | 3.514695 | 11.25296891 |
| 225 | 30 | 5 | 3.494625 | 11.39014633 |
| 63 | 22 | 4 | 3.490895 | 11.41583695 |
| 169 | 65 | 1 | 3.43478 | 11.80986026 |
| 214 | 105 | 13 | 3.407589 | 12.0059821 |
| 190 | 15 | 3 | 3.268415 | 13.0657786 |
| 48 | 204 | 22 | 2.947893 | 15.908072 |
| 185 | 97 | 3 | 2.848601 | 16.918899 |
| 112 | 9 | 2 | 2.727011 | 18.25280512 |
| 232 | 9 | 2 | 2.727011 | 18.25280512 |
| 72 | 76 | 2 | 2.704878 | 18.50771969 |
| 11 | 178 | 8 | 2.509432 | 20.93583779 |
| 279 | 25 | 4 | 2.497684 | 21.09256521 |
| 115 | 34 | 5 | 2.430352 | 22.01613659 |
| 93 | 44 | 6 | 2.26907 | 24.41612784 |
| 224 | 69 | 2 | 2.198724 | 25.55312608 |
| 179 | 10 | 2 | 2.178041 | 25.89862278 |
| 222 | 49 | 1 | 2.176404 | 25.92619164 |
| 144 | 170 | 8 | 2.082036 | 27.57262917 |
| 1 | 67 | 2 | 2.057144 | 28.02636731 |
| 71 | 44 | 1 | 1.794528 | 33.36946226 |
| 67 | 109 | 12 | 1.792701 | 33.41052752 |
| 70 | 131 | 14 | 1.767474 | 33.98357505 |
| 34 | 11 | 2 | 1.743857 | 34.53040856 |
| 194 | 62 | 2 | 1.710373 | 35.32335328 |
| 305 | 62 | 2 | 1.710373 | 35.32335328 |
| 167 | 129 | 6 | 1.63413 | 37.20955663 |
| 171 | 78 | 3 | 1.589409 | 38.37096903 |
| 53 | 20 | 3 | 1.548991 | 39.45761954 |
| 203 | 59 | 2 | 1.508039 | 40.59614356 |
| 125 | 75 | 3 | 1.405913 | 43.61115316 |
| 50 | 12 | 2 | 1.395766 | 43.92525819 |
| 121 | 106 | 5 | 1.281463 | 47.66104276 |
| 207 | 127 | 13 | 1.246161 | 48.89307527 |
| 210 | 94 | 10 | 1.224475 | 49.66957757 |
| 173 | 88 | 4 | 1.191421 | 50.88297792 |
| 14 | 106 | 11 | 1.153401 | 52.32510549 |
| 136 | 5 | 1 | 1.08857 | 54.9058418 |
| 140 | 5 | 1 | 1.08857 | 54.9058418 |
| 165 | 5 | 1 | 1.08857 | 54.9058418 |
| 229 | 5 | 1 | 1.08857 | 54.9058418 |
| 252 | 5 | 1 | 1.08857 | 54.9058418 |
| 15 | 51 | 2 | 0.995887 | 58.88694216 |
| 4 | 33 | 1 | 0.990746 | 59.11861519 |
| 242 | 131 | 7 | 0.976215 | 59.77996664 |
| 81 | 14 | 2 | 0.884079 | 64.21109323 |
| 134 | 14 | 2 | 0.884079 | 64.21109323 |
| 196 | 14 | 2 | 0.884079 | 64.21109323 |
| 76 | 48 | 2 | 0.817038 | 67.71881577 |
| 24 | 6 | 1 | 0.697536 | 74.66837338 |
| 88 | 6 | 1 | 0.697536 | 74.66837338 |
| 96 | 6 | 1 | 0.697536 | 74.66837338 |
| 105 | 6 | 1 | 0.697536 | 74.66837338 |
| 139 | 6 | 1 | 0.697536 | 74.66837338 |
| 142 | 6 | 1 | 0.697536 | 74.66837338 |
| 219 | 6 | 1 | 0.697536 | 74.66837338 |
| 234 | 6 | 1 | 0.697536 | 74.66837338 |
| 266 | 6 | 1 | 0.697536 | 74.66837338 |
| 300 | 6 | 1 | 0.697536 | 74.66837338 |
| 131 | 15 | 2 | 0.695889 | 74.77116667 |
| 61 | 57 | 6 | 0.686281 | 75.37496772 |
| 146 | 149 | 14 | 0.674562 | 76.12115856 |
| 184 | 44 | 2 | 0.593533 | 81.59535961 |
| 77 | 26 | 3 | 0.567748 | 83.46373522 |
| 243 | 90 | 5 | 0.549756 | 84.80724233 |
| 205 | 142 | 13 | 0.479785 | 90.37616832 |
| 235 | 147 | 9 | 0.475043 | 90.77513868 |
| 54 | 25 | 1 | 0.466695 | 91.48466293 |
| 78 | 25 | 1 | 0.466695 | 91.48466293 |
| 244 | 132 | 8 | 0.461907 | 91.89582352 |
| 7 | 72 | 7 | 0.453926 | 92.5881781 |
| 301 | 119 | 11 | 0.449111 | 93.01011434 |
| 47 | 7 | 1 | 0.441783 | 93.65866779 |
| 92 | 7 | 1 | 0.441783 | 93.65866779 |
| 100 | 7 | 1 | 0.441783 | 93.65866779 |
| 236 | 7 | 1 | 0.441783 | 93.65866779 |
| 295 | 7 | 1 | 0.441783 | 93.65866779 |
| 68 | 72 | 4 | 0.439146 | 93.89400517 |
| 218 | 72 | 4 | 0.439146 | 93.89400517 |
| 245 | 96 | 9 | 0.422527 | 95.40061264 |
| 98 | 144 | 13 | 0.409261 | 96.63380036 |
| 82 | 101 | 6 | 0.4088 | 96.67714815 |
| 106 | 24 | 1 | 0.407706 | 96.78019224 |
| 163 | 24 | 1 | 0.407706 | 96.78019224 |
| 195 | 24 | 1 | 0.407706 | 96.78019224 |
| 44 | 115 | 7 | 0.388395 | 98.63165775 |
| 113 | 23 | 1 | 0.350789 | 102.4283332 |
| 161 | 54 | 3 | 0.328866 | 104.7705679 |
| 52 | 83 | 5 | 0.302944 | 107.677896 |
| 174 | 22 | 1 | 0.296225 | 108.4578147 |
| 117 | 68 | 4 | 0.293824 | 108.7393637 |
| 151 | 8 | 1 | 0.270586 | 111.5437565 |
| 182 | 8 | 1 | 0.270586 | 111.5437565 |
| 64 | 251 | 17 | 0.262253 | 112.5867005 |
| 25 | 67 | 4 | 0.260992 | 112.7463084 |
| 212 | 37 | 2 | 0.25891 | 113.0109209 |
| 45 | 21 | 1 | 0.244349 | 114.9003266 |
| 87 | 21 | 1 | 0.244349 | 114.9003266 |
| 94 | 21 | 1 | 0.244349 | 114.9003266 |
| 247 | 50 | 3 | 0.187528 | 123.0217539 |
| 154 | 31 | 3 | 0.186555 | 123.1728561 |
| 204 | 31 | 3 | 0.186555 | 123.1728561 |
| 57 | 9 | 1 | 0.155763 | 128.2212504 |
| 198 | 9 | 1 | 0.155763 | 128.2212504 |
| 38 | 19 | 1 | 0.150344 | 129.1682613 |
| 126 | 63 | 4 | 0.145636 | 130.0071775 |
| 227 | 106 | 9 | 0.114675 | 135.9533115 |
| 137 | 18 | 1 | 0.109295 | 137.0754076 |
| 43 | 185 | 13 | 0.094348 | 140.3632621 |
| 241 | 10 | 1 | 0.080407 | 143.6981855 |
| 90 | 17 | 1 | 0.073146 | 145.5599846 |
| 217 | 17 | 1 | 0.073146 | 145.5599846 |
| 186 | 115 | 8 | 0.072953 | 145.6108044 |
| 147 | 159 | 13 | 0.070349 | 146.3033979 |
| 69 | 96 | 8 | 0.069613 | 146.5016486 |
| 21 | 87 | 6 | 0.065438 | 147.6482552 |
| 246 | 109 | 9 | 0.063039 | 148.3248525 |
| 66 | 31 | 2 | 0.060431 | 149.0761067 |
| 178 | 127 | 9 | 0.052184 | 151.5715783 |
| 123 | 16 | 1 | 0.042812 | 154.6748631 |
| 254 | 30 | 2 | 0.038956 | 156.0542162 |
| 199 | 23 | 2 | 0.037753 | 156.499015 |
| 122 | 71 | 5 | 0.034052 | 157.915145 |
| 75 | 11 | 1 | 0.033757 | 158.0314725 |
| 111 | 11 | 1 | 0.033757 | 158.0314725 |
| 215 | 11 | 1 | 0.033757 | 158.0314725 |
| 221 | 57 | 4 | 0.029685 | 159.6933827 |
| 132 | 36 | 3 | 0.025975 | 161.3128824 |
| 79 | 74 | 6 | 0.025025 | 161.7463884 |
| 200 | 15 | 1 | 0.019454 | 164.4786283 |
| 119 | 56 | 4 | 0.018318 | 165.0828647 |
| 213 | 56 | 4 | 0.018318 | 165.0828647 |
| 85 | 83 | 6 | 0.018308 | 165.0881547 |
| 95 | 204 | 16 | 0.014458 | 167.2939898 |
| 99 | 166 | 13 | 0.01051 | 169.8939747 |
| 166 | 205 | 16 | 0.009944 | 170.3049558 |
| 127 | 95 | 7 | 0.008721 | 171.235585 |
| 97 | 12 | 1 | 0.008641 | 171.2984018 |
| 202 | 12 | 1 | 0.008641 | 171.2984018 |
| 209 | 25 | 2 | 0.005093 | 174.4743506 |
| 2 | 14 | 1 | 0.004564 | 175.0359932 |
| 172 | 14 | 1 | 0.004564 | 175.0359932 |
| 216 | 14 | 1 | 0.004564 | 175.0359932 |
| 6 | 77 | 6 | 0.003202 | 176.652057 |
| 107 | 52 | 4 | 0.00037 | 182.1595807 |
| 58 | 26 | 2 | 0.000185 | 182.9936248 |
| 46 | 13 | 1 | 9.23E-05 | 183.5820208 |
