# Supplementary material for: Integrated network analysis reveals potentially novel molecular mechanisms and therapeutic targets of refractory epilepsies
Source: PLoS One. 2017 Apr 7;12(4):e0174964. doi: 10.1371/journal.pone.0174964 (PMC5384674; doi:10.1371/journal.pone.0174964)
Supplement: S6 Table — (DOCX) [file pone.0174964.s006.docx]

**S6 Table. Primary 42 modules filtered by RR values.**

| **Module** | **Number of seed genes** | **Number of Proteins** | **Ratio** | **Relative Risk** |
| --- | --- | --- | --- | --- |
| 145 | 7 | 7 | 1 | 13.12052 |
| 83 | 2 | 5 | 0.4 | 5.248208 |
| 129 | 2 | 6 | 0.333333 | 4.373507 |
| 150 | 2 | 6 | 0.333333 | 4.373507 |
| 155 | 40 | 141 | 0.283688 | 3.722134 |
| 188 | 12 | 43 | 0.27907 | 3.661541 |
| 271 | 4 | 16 | 0.25 | 3.28013 |
| 208 | 8 | 34 | 0.235294 | 3.087181 |
| 112 | 2 | 9 | 0.222222 | 2.915671 |
| 232 | 2 | 9 | 0.222222 | 2.915671 |
| 197 | 11 | 50 | 0.22 | 2.886515 |
| 27 | 4 | 19 | 0.210526 | 2.762215 |
| 136 | 1 | 5 | 0.2 | 2.624104 |
| 140 | 1 | 5 | 0.2 | 2.624104 |
| 165 | 1 | 5 | 0.2 | 2.624104 |
| 179 | 2 | 10 | 0.2 | 2.624104 |
| 190 | 3 | 15 | 0.2 | 2.624104 |
| 229 | 1 | 5 | 0.2 | 2.624104 |
| 252 | 1 | 5 | 0.2 | 2.624104 |
| 65 | 36 | 184 | 0.195652 | 2.567058 |
| 80 | 10 | 52 | 0.192308 | 2.523177 |
| 26 | 4 | 21 | 0.190476 | 2.499147 |
| 34 | 2 | 11 | 0.181818 | 2.385549 |
| 63 | 4 | 22 | 0.181818 | 2.385549 |
| 114 | 9 | 53 | 0.169811 | 2.228013 |
| 24 | 1 | 6 | 0.166667 | 2.186754 |
| 50 | 2 | 12 | 0.166667 | 2.186754 |
| 88 | 1 | 6 | 0.166667 | 2.186754 |
| 96 | 1 | 6 | 0.166667 | 2.186754 |
| 105 | 1 | 6 | 0.166667 | 2.186754 |
| 139 | 1 | 6 | 0.166667 | 2.186754 |
| 142 | 1 | 6 | 0.166667 | 2.186754 |
| 219 | 1 | 6 | 0.166667 | 2.186754 |
| 225 | 5 | 30 | 0.166667 | 2.186754 |
| 234 | 1 | 6 | 0.166667 | 2.186754 |
| 253 | 6 | 36 | 0.166667 | 2.186754 |
| 266 | 1 | 6 | 0.166667 | 2.186754 |
| 300 | 1 | 6 | 0.166667 | 2.186754 |
| 279 | 4 | 25 | 0.16 | 2.099283 |
| 230 | 7 | 45 | 0.155556 | 2.04097 |
| 53 | 3 | 20 | 0.15 | 1.968078 |
| 37 | 45 | 305 | 0.147541 | 1.935815 |
