# Supplementary material for: Integrated network analysis reveals potentially novel molecular mechanisms and therapeutic targets of refractory epilepsies
Source: PLoS One. 2017 Apr 7;12(4):e0174964. doi: 10.1371/journal.pone.0174964 (PMC5384674; doi:10.1371/journal.pone.0174964)
Supplement: S7 Table — We listed the smallest p-value terms of each module. If the smallest p-value >0.05, we leave it blank. (DOCX) [file pone.0174964.s007.docx]

# S7 Table. GO enrichment analysis of all 42 modules. We listed the smallest p-value terms of each module. If the smallest p-value >0.05, we leave it blank.

| **Module** | **BP** | | **CC** | | **MF** | |
| --- | --- | --- | --- | --- | --- | --- |
|  | **GO**  **term** | **p-value** | **GO**  **term** | **p-value** | **GO**  **term** | **p-value** |
| M24 | zinc ion transport | 8.60E-13 | integral to membrane | 1.94E-02 | zinc ion transmembrane transporter activity | 1.77E-11 |
| M26 | sensory perception of sound | 5.26E-19 | stereocilium | 2.24E-12 |  |  |
| M27 | fusion of sperm to egg plasma membrane | 5.73E-06 | lysosome | 3.32E-09 | sulfuric ester hydrolase activity | 5.28E-29 |
| M34 |  |  |  |  | GTPase regulator activity | 2.62E-05 |
| M37 | oxidative phosphorylation | 0.00E+00 | mitochondrial membrane part | 0.00E+00 | hydrogen ion transmembrane transporter activity | 0.00E+00 |
| M50 |  |  |  |  |  |  |
| M53 | indolalkylamine metabolic process | 4.28E-19 | cytosol | 2.62E-07 | kynurenine-oxoglutarate transaminase activity | 1.22E-07 |
| M63 | prosthetic group metabolic process | 2.90E-06 | chloride channel complex | 1.99E-05 | extracellular-glycine-gated ion channel activity | 2.98E-10 |
| M65 | ion transport | 2.29E-43 | calcium channel complex | 9.34E-47 | gated channel activity | 3.87E-53 |
| M80 | regulation of TOR signaling cascade | 8.69E-13 | cytosol | 1.31E-07 | protein serine/threonine kinase activity | 4.76E-05 |
| M83 | calcium-independent cell-matrix adhesion | 2.75E-02 | polycystin complex | 1.55E-02 |  |  |
| M88 | sex determination | 2.40E-03 |  |  | transcription factor activity | 1.59E-02 |
| M96 | immune system process | 9.04E-04 |  |  | tumor necrosis factor receptor activity | 4.54E-02 |
| M105 | negative regulation of mitotic prometaphase | 2.26E-02 |  |  | GABA-B receptor activity | 4.31E-02 |
| M112 | cerebral cortex GABAergic interneuron fate commitment | 2.37E-05 |  |  | transcription regulator activity | 1.64E-05 |
| M114 | regulation of fatty acid oxidation | 4.68E-22 | AMP-activated protein kinase complex | 1.90E-11 | protein serine/threonine kinase activity | 2.68E-15 |
| M129 | digestion | 2.62E-02 | apical lamina of hyaline layer | 7.88E-03 | armadillo repeat domain binding | 2.67E-02 |
| M136 | chaperone mediated protein folding requiring cofactor | 4.62E-05 | endoplasmic reticulum lumen | 6.73E-03 | unfolded protein binding | 1.83E-02 |
| M139 | thalamus development | 4.75E-02 |  |  | zinc ion binding | 2.21E-03 |
| M140 |  |  | ER-Golgi intermediate compartment membrane | 5.24E-04 |  |  |
| M142 | regulation of transcription from RNA polymerase II promoter involved in spinal cord association neuron specification | 1.78E-02 | transcription factor complex | 2.66E-02 |  |  |
| M145 | negative regulation of cyclase activity | 8.16E-10 | integral to plasma membrane | 2.81E-07 | glutamate receptor activity | 8.27E-19 |
| M150 |  |  |  |  |  |  |
| M155 | ion transport | 4.02E-69 | ion channel complex | 6.63E-83 | gated channel activity | 6.01E-98 |
| M165 |  |  |  |  | GTPase activator activity | 1.61E-02 |
| M179 |  |  |  |  | bent DNA binding | 3.96E-02 |
| M188 | synaptic transmission, cholinergic | 1.10E-28 | nicotinic acetylcholine-gated receptor-channel complex | 5.90E-38 | nicotinic acetylcholine-activated cation-selective channel activity | 9.95E-35 |
| M190 | regulation of transcription, DNA-dependent | 7.83E-09 | nucleus | 9.54E-06 | nucleic acid binding | 4.00E-05 |
| M197 | membrane invagination | 1.35E-09 | coated pit | 5.83E-07 | lipoprotein binding | 1.68E-09 |
| M208 | muscle organ development | 3.62E-09 | dystrophin-associated glycoprotein complex | 1.09E-42 | calcium ion binding | 8.94E-04 |
| M219 | cell-cell adhesion | 2.12E-04 | integral to plasma membrane | 1.29E-04 | integrin binding | 3.25E-03 |
| M225 | neurotransmitter transport | 3.98E-16 | synapse | 1.02E-19 | Rab geranylgeranyltransferase activity | 2.62E-06 |
| M229 | regulation of cell shape | 5.79E-05 | cytoplasmic membrane-bounded vesicle | 9.66E-03 | D3 dopamine receptor binding | 8.29E-03 |
| M230 | protein localization | 6.44E-15 | endosome | 3.62E-29 |  |  |
| M232 | proteolysis | 1.50E-09 | integral to membrane | 7.26E-03 | metalloendopeptidase activity | 4.37E-16 |
| M234 | regulation of Rap GTPase activity | 5.73E-03 | postsynaptic density | 2.09E-05 |  |  |
| M252 | activation of store-operated calcium channel activity | 6.16E-06 |  |  | calcium ion binding | 5.61E-03 |
| M253 | actin filament capping | 1.03E-11 | spectrin | 4.05E-16 | structural constituent of cytoskeleton | 1.86E-10 |
| M266 | positive regulation of heart rate in baroreceptor response to decreased systemic arterial blood pressure | 1.33E-02 | anchored to membrane | 5.00E-02 | adenylate cyclase binding | 2.95E-02 |
| M271 | neurotransmitter transport | 1.58E-23 | integral to plasma membrane | 2.83E-14 | neurotransmitter:sodium symporter activity | 1.21E-33 |
| M279 | cell division | 1.25E-07 | septin cytoskeleton | 5.91E-23 | GTP binding | 4.15E-09 |
| M300 | transcription from mitochondrial promoter | 1.97E-04 | nucleoid | 1.71E-06 | RNA methyltransferase activity | 1.35E-03 |
