# Supplementary material for: Integrated network analysis reveals potentially novel molecular mechanisms and therapeutic targets of refractory epilepsies
Source: PLoS One. 2017 Apr 7;12(4):e0174964. doi: 10.1371/journal.pone.0174964 (PMC5384674; doi:10.1371/journal.pone.0174964)
Supplement: S8 Table — (DOCX) [file pone.0174964.s008.docx]

**S8 Table. Combined score of module pair.**

| **M1** | **M2** | **GO-BP** | **GO-CC** | **GO-MF** | **Pathway** | **Combined_score** |
| --- | --- | --- | --- | --- | --- | --- |
| M65 | M155 | 0.615159 | 0.55713 | 0.8142115 | 0.36384 | 0.513 |
| M279 | M155 | 0.005719 | 0.01728 | 0.323238 | 0.75281 | 0.43411 |
| M80 | M114 | 0.09662 | 0.23989 | 0.644058 | 0.51532 | 0.42109 |
| M65 | M188 | 0.357718 | 0.3559 | 0.4900252 | 0.26795 | 0.33458 |
| M271 | M225 | 0.446168 | 0.17534 | 0.3231379 | 0.33153 | 0.32321 |
| M271 | M24 | 0.074711 | 0.4227 | 0.3470001 | 0.31734 | 0.29941 |
| M188 | M155 | 0.305668 | 0.38634 | 0.4168068 | 0.17819 | 0.2739 |
| M63 | M197 | 0 | 0.0169 | 0.008986 | 0.49977 | 0.2542 |
| M65 | M225 | 0.230042 | 0.32949 | 0.109584 | 0.23824 | 0.23064 |
| M225 | M155 | 0.388594 | 0.2206 | 0.0945949 | 0.17706 | 0.20583 |
| M65 | M279 | 0 | 0.03205 | 0.2197771 | 0.31996 | 0.20195 |
| M225 | M188 | 0.277498 | 0.31985 | 0.0744317 | 0.16974 | 0.19683 |
| M88 | M190 | 0.566304 | 0 | 0.5383489 | 0 | 0.18411 |
| M271 | M155 | 0.209741 | 0.5111 | 0.1763289 | 0.04743 | 0.17324 |
| M88 | M112 | 0.239036 | 0 | 0.6943553 | 0 | 0.15557 |
| M271 | M219 | 0 | 0.92897 | 0 | 0 | 0.15483 |
| M65 | M24 | 0.341693 | 0.26136 | 0.2828215 | 0.01163 | 0.15346 |
| M24 | M155 | 0.373849 | 0.25849 | 0.2652362 | 0.00725 | 0.15322 |
| M63 | M188 | 0.077818 | 0.54622 | 0.268388 | 0.00289 | 0.15018 |
| M65 | M271 | 0.111432 | 0.30563 | 0.1913886 | 0.06652 | 0.13467 |
| M34 | M165 | 0 | 0 | 0.7574075 | 0 | 0.12623 |
| M271 | M145 | 0.033427 | 0.68345 | 0 | 0 | 0.11948 |
| M190 | M112 | 0.263083 | 0 | 0.4374209 | 0 | 0.11675 |
| M225 | M145 | 0.260295 | 0.42805 | 0 | 0 | 0.11472 |
| M24 | M225 | 0.10267 | 0.12396 | 0.1177006 | 0.11375 | 0.11427 |
| M63 | M155 | 0.040637 | 0.38469 | 0.1870994 | 0.02125 | 0.1127 |
| M24 | M230 | 0.084254 | 0.46063 | 0 | 0.04174 | 0.11168 |
| M26 | M197 | 0.029919 | 0.08992 | 0 | 0.18113 | 0.11054 |
| M188 | M145 | 0.256488 | 0.35237 | 0.0361651 | 0 | 0.1075 |
| M24 | M232 | 0 | 0.64289 | 0 | 0 | 0.10715 |
| M219 | M145 | 0 | 0.63041 | 0 | 0 | 0.10507 |
| M65 | M63 | 0.041726 | 0.3396 | 0.2303252 | 0.00118 | 0.10253 |
| M253 | M155 | 0 | 0.13499 | 0.0011544 | 0.15902 | 0.1022 |
| M65 | M145 | 0.138764 | 0.4003 | 0.0673036 | 0 | 0.10106 |
| M155 | M145 | 0.136455 | 0.4614 | 0.005994 | 0 | 0.10064 |
| M230 | M197 | 0.249197 | 0.34912 | 0 | 0 | 0.09972 |
| M271 | M188 | 0.052973 | 0.21685 | 0.1282971 | 0.05939 | 0.09605 |
| M232 | M219 | 0 | 0.49098 | 0.0811055 | 0 | 0.09535 |
| M63 | M234 | 0 | 0.57197 | 0 | 0 | 0.09533 |
| M65 | M232 | 0.00849 | 0.23996 | 0 | 0.09894 | 0.09088 |
| M80 | M53 | 0 | 0.53862 | 0 | 0 | 0.08977 |
| M279 | M188 | 0 | 0.00688 | 0.081433 | 0.14262 | 0.08603 |
| M37 | M24 | 0.096146 | 0.0387 | 0.2948235 | 0.02741 | 0.08532 |
| M37 | M271 | 0.091572 | 0.02511 | 0.2842231 | 0.03415 | 0.08389 |
| M271 | M26 | 0 | 0.1326 | 0 | 0.12217 | 0.08319 |
| M230 | M225 | 0.302521 | 0.12577 | 0 | 0.02171 | 0.08224 |
| M234 | M188 | 0.022996 | 0.46214 | 0 | 0 | 0.08086 |
| M279 | M225 | 0 | 0 | 0 | 0.16121 | 0.08061 |
| M219 | M155 | 0 | 0.48001 | 0 | 0 | 0.08 |
| M24 | M188 | 0.13051 | 0.08895 | 0.1744202 | 0.02358 | 0.07744 |
| M65 | M230 | 0.147189 | 0.07838 | 0 | 0.07475 | 0.07497 |
| M271 | M232 | 0 | 0.44827 | 0 | 0 | 0.07471 |
| M37 | M155 | 0.200403 | 0.02827 | 0.1855444 | 0.00936 | 0.07372 |
| M63 | M225 | 0.059449 | 0.34386 | 0.0321999 | 0 | 0.07259 |
| M253 | M208 | 0.010013 | 0.08004 | 0.344811 | 0 | 0.07248 |
| M24 | M219 | 0 | 0.43353 | 0 | 0 | 0.07225 |
| M234 | M225 | 0.025929 | 0.38473 | 0 | 0 | 0.06844 |
| M234 | M145 | 0 | 0.4066 | 0 | 0 | 0.06777 |
| M252 | M208 | 0 | 0 | 0.4031536 | 0 | 0.06719 |
| M229 | M225 | 0.012861 | 0.37781 | 0 | 0 | 0.06511 |
| M271 | M253 | 0 | 0.22111 | 0.0166951 | 0.04704 | 0.06316 |
| M65 | M37 | 0.114054 | 0.04183 | 0.2020435 | 0.00695 | 0.06313 |
| M271 | M230 | 0.17743 | 0.02272 | 0 | 0.052 | 0.05936 |
| M83 | M266 | 0 | 0.35212 | 0 | 0 | 0.05869 |
| M63 | M145 | 0.059916 | 0.28616 | 0.0057915 | 0 | 0.05865 |
| M63 | M53 | 0.099295 | 0 | 0.1912609 | 0.01916 | 0.05801 |
| M225 | M197 | 0.088933 | 0.25509 | 0 | 0 | 0.05734 |
| M232 | M139 | 0 | 0 | 0.3372366 | 0 | 0.05621 |
| M229 | M197 | 0.021722 | 0.30713 | 0 | 0 | 0.05481 |
| M65 | M234 | 0 | 0.32817 | 0 | 0 | 0.0547 |
| M53 | M37 | 0.033067 | 0.10293 | 0.0305668 | 0.05275 | 0.05414 |
| M26 | M253 | 0.017235 | 0.08188 | 0 | 0.07497 | 0.054 |
| M65 | M219 | 0 | 0.29996 | 0 | 0.00462 | 0.0523 |
| M24 | M145 | 0.005159 | 0.27872 | 0.019915 | 0 | 0.05063 |
| M37 | M225 | 0.120921 | 0.02382 | 0.1154544 | 0.01426 | 0.0505 |
| M53 | M114 | 0.113413 | 0.16794 | 0.0093273 | 0 | 0.04845 |
| M65 | M253 | 0.004365 | 0.12451 | 0.0110488 | 0.04978 | 0.04821 |
| M279 | M253 | 0 | 0.26693 | 0.0222889 | 0 | 0.0482 |
| M65 | M197 | 0.053433 | 0.20301 | 0.0041063 | 0.00801 | 0.04743 |
| M230 | M219 | 0 | 0.02482 | 0 | 0.0849 | 0.04659 |
| M300 | M136 | 0 | 0.27906 | 0 | 0 | 0.04651 |
| M232 | M145 | 0 | 0.277 | 0 | 0 | 0.04617 |
| M230 | M155 | 0.233299 | 0.01944 | 0 | 0.00648 | 0.04536 |
| M27 | M232 | 0.19661 | 0 | 0.0754653 | 0 | 0.04535 |
| M234 | M155 | 0 | 0.26406 | 0 | 0 | 0.04401 |
| M80 | M230 | 0.122195 | 0.10495 | 0 | 0.01189 | 0.0438 |
| M232 | M155 | 0 | 0.25495 | 0 | 0 | 0.04249 |
| M266 | M188 | 0.232274 | 0 | 0.0169266 | 0 | 0.04153 |
| M37 | M230 | 0.114796 | 0.05966 | 0 | 0.02451 | 0.04133 |
| M300 | M190 | 0 | 0 | 0.2421458 | 0 | 0.04036 |
| M300 | M114 | 0 | 0.04027 | 0 | 0.06715 | 0.04029 |
| M37 | M300 | 0.023426 | 0.21057 | 0 | 0 | 0.039 |
| M88 | M300 | 0 | 0 | 0.2325818 | 0 | 0.03876 |
| M24 | M197 | 0.046821 | 0.18482 | 0 | 0 | 0.03861 |
| M27 | M197 | 0.034541 | 0.15893 | 0 | 0.01261 | 0.03855 |
| M230 | M229 | 0 | 0.23062 | 0 | 0 | 0.03844 |
| M208 | M197 | 0.042366 | 0.09493 | 0.0875721 | 0 | 0.03748 |
| M80 | M65 | 0.036496 | 0.04799 | 0.0179889 | 0.03902 | 0.03659 |
| M80 | M197 | 0.06142 | 0.10997 | 0.0444887 | 0 | 0.03598 |
| M83 | M26 | 0 | 0.21407 | 0 | 0 | 0.03568 |
| M253 | M219 | 0 | 0.20917 | 0 | 0 | 0.03486 |
| M37 | M188 | 0.045076 | 0.00894 | 0.1247819 | 0.00796 | 0.03378 |
| M266 | M26 | 0 | 0.20181 | 0 | 0 | 0.03363 |
| M26 | M225 | 0.070474 | 0.05735 | 0 | 0.02451 | 0.03356 |
| M53 | M300 | 0.024638 | 0.17669 | 0 | 0 | 0.03356 |
| M253 | M145 | 0 | 0.20105 | 0 | 0 | 0.03351 |
| M219 | M188 | 0 | 0.20073 | 0 | 0 | 0.03345 |
| M26 | M145 | 0.09151 | 0.10651 | 0 | 0 | 0.033 |
| M208 | M188 | 0.10798 | 0.08973 | 0 | 0 | 0.03295 |
| M197 | M145 | 0.023595 | 0.17304 | 0 | 0 | 0.03277 |
| M65 | M208 | 0.017909 | 0.16553 | 0.0037128 | 0 | 0.03119 |
| M279 | M114 | 0.008211 | 0 | 0.1761019 | 0 | 0.03072 |
| M80 | M253 | 0.118441 | 0.03272 | 0.0329668 | 0 | 0.03069 |
| M63 | M37 | 0.026748 | 0.04274 | 0.0703982 | 0.01453 | 0.03058 |
| M271 | M197 | 0.054172 | 0.1281 | 0 | 0 | 0.03038 |
| M26 | M112 | 0.180912 | 0 | 0 | 0 | 0.03015 |
| M26 | M219 | 0.039333 | 0.13675 | 0 | 0 | 0.02935 |
| M225 | M219 | 0 | 0.17403 | 0 | 0 | 0.02901 |
| M53 | M188 | 0.10293 | 0 | 0.0368415 | 0.01082 | 0.02871 |
| M197 | M155 | 0.06597 | 0.1054 | 6.09E-04 | 0 | 0.02866 |
| M225 | M208 | 0.006604 | 0.1589 | 0 | 0 | 0.02758 |
| M230 | M114 | 0.033467 | 0.04745 | 0 | 0.02689 | 0.02693 |
| M252 | M197 | 0.016528 | 0 | 0.142299 | 0 | 0.02647 |
| M253 | M225 | 0.003046 | 0.08972 | 0.0152144 | 0.01573 | 0.02586 |
| M80 | M279 | 0.034305 | 0.00532 | 0.1124979 | 0 | 0.02535 |
| M197 | M188 | 0.054363 | 0.05568 | 0.0077604 | 0.00985 | 0.02456 |
| M300 | M112 | 0 | 0 | 0.1457932 | 0 | 0.0243 |
| M253 | M24 | 0.004389 | 0.07763 | 0 | 0.01906 | 0.0232 |
| M65 | M26 | 0.038741 | 0.10032 | 0 | 0 | 0.02318 |
| M253 | M197 | 0.026455 | 0.09427 | 0.0179293 | 0 | 0.02311 |
| M37 | M114 | 0.055073 | 0.02889 | 0.0031757 | 0.01695 | 0.023 |
| M65 | M229 | 0 | 0.1376 | 0 | 0 | 0.02293 |
| M234 | M230 | 0.086001 | 0.04925 | 0 | 0 | 0.02254 |
| M53 | M271 | 0.007196 | 0 | 0.0013631 | 0.04176 | 0.02231 |
| M219 | M197 | 0 | 0.1307 | 0 | 0 | 0.02178 |
| M208 | M155 | 0.006627 | 0.12287 | 0 | 0 | 0.02158 |
| M271 | M208 | 0 | 0.12859 | 0 | 0 | 0.02143 |
| M219 | M208 | 0 | 0.12813 | 0 | 0 | 0.02135 |
| M80 | M37 | 0.023365 | 0.06468 | 0 | 0.01243 | 0.02089 |
| M208 | M145 | 0.008622 | 0.11566 | 0 | 0 | 0.02071 |
| M26 | M155 | 0.039009 | 0.08481 | 0 | 0 | 0.02064 |
| M271 | M234 | 0 | 0.12373 | 0 | 0 | 0.02062 |
| M88 | M208 | 0.122751 | 0 | 0 | 0 | 0.02046 |
| M53 | M197 | 0.007977 | 0.09639 | 0.0168393 | 0 | 0.0202 |
| M234 | M219 | 0 | 0.11947 | 0 | 0 | 0.01991 |
| M234 | M208 | 0 | 0.11839 | 0 | 0 | 0.01973 |
| M26 | M232 | 0 | 0.11796 | 0 | 0 | 0.01966 |
| M229 | M145 | 0.117769 | 0 | 0 | 0 | 0.01963 |
| M279 | M271 | 0 | 0 | 0.0100043 | 0.03545 | 0.01939 |
| M252 | M24 | 0.064634 | 0 | 0.050414 | 0 | 0.01917 |
| M232 | M197 | 0.015765 | 0.0981 | 0 | 0 | 0.01898 |
| M88 | M253 | 0.110798 | 0 | 0 | 0 | 0.01847 |
| M80 | M190 | 0.046704 | 0.06256 | 0 | 0 | 0.01821 |
| M80 | M145 | 0.073681 | 0.03538 | 0 | 0 | 0.01818 |
| M26 | M24 | 0 | 0.10817 | 0 | 0 | 0.01803 |
| M27 | M219 | 0.107778 | 0 | 0 | 0 | 0.01796 |
| M190 | M114 | 0.042768 | 0.06259 | 0 | 0 | 0.01756 |
| M63 | M24 | 0 | 0.05047 | 0.0518777 | 0 | 0.01706 |
| M230 | M188 | 0.092715 | 0.00953 | 0 | 0 | 0.01704 |
| M252 | M145 | 0.013562 | 0 | 0.0864728 | 0 | 0.01667 |
| M80 | M300 | 0 | 0.09929 | 0 | 0 | 0.01655 |
| M24 | M208 | 0 | 0.09815 | 0 | 0 | 0.01636 |
| M26 | M188 | 0.065181 | 0.0324 | 0 | 0 | 0.01626 |
| M63 | M271 | 0 | 0.06096 | 0.0363036 | 0 | 0.01621 |
| M65 | M252 | 0.096964 | 0 | 0 | 0 | 0.01616 |
| M229 | M208 | 0.02197 | 0.07218 | 0 | 0 | 0.01569 |
| M253 | M234 | 0 | 0.09141 | 0 | 0 | 0.01524 |
| M53 | M230 | 0 | 0.09132 | 0 | 0 | 0.01522 |
| M37 | M27 | 0.004431 | 0.00555 | 0.0057833 | 0.02372 | 0.01449 |
| M53 | M27 | 0.004484 | 0 | 0.0169861 | 0.02176 | 0.01446 |
| M83 | M80 | 0.086373 | 0 | 0 | 0 | 0.0144 |
| M190 | M145 | 0.086033 | 0 | 0 | 0 | 0.01434 |
| M197 | M114 | 0.04488 | 0.02842 | 0.0111118 | 0 | 0.01407 |
| M26 | M208 | 0.032737 | 0.0516 | 0 | 0 | 0.01406 |
| M232 | M225 | 0 | 0.08288 | 0 | 0 | 0.01381 |
| M27 | M139 | 0 | 0 | 0.0824472 | 0 | 0.01374 |
| M63 | M27 | 0.038565 | 0 | 0.0231863 | 0.00581 | 0.0132 |
| M65 | M114 | 0.010992 | 0.00836 | 0.0215522 | 0.01254 | 0.01309 |
| M37 | M190 | 0 | 0.07844 | 0 | 0 | 0.01307 |
| M80 | M112 | 0.076379 | 0 | 0 | 0 | 0.01273 |
| M232 | M188 | 0 | 0.07543 | 0 | 0 | 0.01257 |
| M80 | M208 | 0.031734 | 0.04219 | 0 | 0 | 0.01232 |
| M232 | M208 | 0 | 0.07174 | 0 | 0 | 0.01196 |
| M37 | M197 | 0.035235 | 0.03598 | 4.66E-04 | 0 | 0.01195 |
| M53 | M253 | 0 | 0.04527 | 0.0261002 | 0 | 0.01189 |
| M88 | M232 | 0.071125 | 0 | 0 | 0 | 0.01185 |
| M63 | M208 | 0 | 0.07077 | 0 | 0 | 0.0118 |
| M88 | M145 | 0.070476 | 0 | 0 | 0 | 0.01175 |
| M253 | M188 | 0.009462 | 0.05635 | 0 | 0.0015 | 0.01172 |
| M80 | M219 | 0 | 0 | 0.0702 | 0 | 0.0117 |
| M53 | M225 | 0.001102 | 0.05248 | 0.0153388 | 0 | 0.01149 |
| M145 | M112 | 0.068883 | 0 | 0 | 0 | 0.01148 |
| M37 | M232 | 0.006819 | 0.0556 | 0.0038634 | 0 | 0.01105 |
| M83 | M197 | 0.036646 | 0.02654 | 0 | 0 | 0.01053 |
| M230 | M190 | 0 | 0.06264 | 0 | 0 | 0.01044 |
| M63 | M139 | 0 | 0 | 0.0620444 | 0 | 0.01034 |
| M208 | M112 | 0.061395 | 0 | 0 | 0 | 0.01023 |
| M232 | M114 | 0.052108 | 0 | 0.0074212 | 0 | 0.00992 |
| M230 | M145 | 0.035022 | 0.02385 | 0 | 0 | 0.00981 |
| M88 | M80 | 0.058746 | 0 | 0 | 0 | 0.00979 |
| M197 | M112 | 0.057463 | 0 | 0 | 0 | 0.00958 |
| M65 | M53 | 4.94E-04 | 0.04862 | 0.0083413 | 0 | 0.00958 |
| M83 | M112 | 0.057366 | 0 | 0 | 0 | 0.00956 |
| M63 | M232 | 0.028346 | 0 | 0.0278157 | 0 | 0.00936 |
| M63 | M219 | 0 | 0.05304 | 0 | 0 | 0.00884 |
| M279 | M234 | 0 | 0.05289 | 0 | 0 | 0.00881 |
| M253 | M232 | 0 | 0.0524 | 0 | 0 | 0.00873 |
| M24 | M234 | 0 | 0.05127 | 0 | 0 | 0.00854 |
| M252 | M155 | 0.045351 | 0 | 0.0025744 | 0 | 0.00799 |
| M88 | M26 | 0.047503 | 0 | 0 | 0 | 0.00792 |
| M230 | M208 | 0 | 0.04672 | 0 | 0 | 0.00779 |
| M253 | M229 | 0.046515 | 0 | 0 | 0 | 0.00775 |
| M80 | M188 | 0.041064 | 0.0035 | 0 | 0 | 0.00743 |
| M188 | M112 | 0.044467 | 0 | 0 | 0 | 0.00741 |
| M27 | M188 | 0 | 0 | 0 | 0.01456 | 0.00728 |
| M80 | M129 | 0.042254 | 0 | 0 | 0 | 0.00704 |
| M300 | M230 | 0 | 0.04185 | 0 | 0 | 0.00698 |
| M88 | M188 | 0.040687 | 0 | 0 | 0 | 0.00678 |
| M234 | M197 | 0 | 0.03906 | 0 | 0 | 0.00651 |
| M279 | M145 | 0 | 0.039 | 0 | 0 | 0.0065 |
| M88 | M114 | 0.038762 | 0 | 0 | 0 | 0.00646 |
| M279 | M197 | 0 | 0.02358 | 0.0151457 | 0 | 0.00645 |
| M26 | M234 | 0 | 0.03866 | 0 | 0 | 0.00644 |
| M145 | M114 | 0.038427 | 0 | 0 | 0 | 0.0064 |
| M300 | M197 | 0 | 0.03797 | 0 | 0 | 0.00633 |
| M80 | M225 | 0.012962 | 0.02339 | 0 | 0 | 0.00606 |
| M80 | M234 | 0.035687 | 0 | 0 | 0 | 0.00595 |
| M208 | M114 | 0 | 0.03531 | 0 | 0 | 0.00589 |
| M112 | M105 | 0.033791 | 0 | 0 | 0 | 0.00563 |
| M88 | M27 | 0.033709 | 0 | 0 | 0 | 0.00562 |
| M63 | M279 | 0 | 0 | 0.0332014 | 0 | 0.00553 |
| M225 | M112 | 0.032908 | 0 | 0 | 0 | 0.00548 |
| M279 | M208 | 0 | 0.03104 | 0 | 0 | 0.00517 |
| M83 | M65 | 0 | 0.03023 | 0 | 0 | 0.00504 |
| M300 | M208 | 0 | 0.03006 | 0 | 0 | 0.00501 |
| M27 | M230 | 0.028874 | 0 | 0 | 0 | 0.00481 |
| M63 | M300 | 0 | 0.02864 | 0 | 0 | 0.00477 |
| M37 | M219 | 0 | 0.02827 | 0 | 0 | 0.00471 |
| M208 | M190 | 0 | 0.02784 | 0 | 0 | 0.00464 |
| M88 | M197 | 0.027599 | 0 | 0 | 0 | 0.0046 |
| M53 | M208 | 0 | 0.02755 | 0 | 0 | 0.00459 |
| M37 | M208 | 0 | 0.02658 | 0 | 0 | 0.00443 |
| M63 | M26 | 0.012885 | 0.01328 | 0 | 0 | 0.00436 |
| M279 | M230 | 0.023243 | 0.00121 | 0 | 0 | 0.00408 |
| M80 | M155 | 0.015052 | 0.00717 | 0.0020942 | 0 | 0.00405 |
| M279 | M26 | 0 | 0.02398 | 0 | 0 | 0.004 |
| M37 | M234 | 0 | 0.02378 | 0 | 0 | 0.00396 |
| M27 | M26 | 0.023551 | 0 | 0 | 0 | 0.00393 |
| M253 | M114 | 0 | 0.00634 | 0.0170437 | 0 | 0.0039 |
| M300 | M253 | 0 | 0.02301 | 0 | 0 | 0.00383 |
| M188 | M114 | 0.021766 | 0 | 0 | 0 | 0.00363 |
| M53 | M139 | 0.021349 | 0 | 0 | 0 | 0.00356 |
| M53 | M232 | 0.009838 | 0 | 0.0113473 | 0 | 0.00353 |
| M252 | M188 | 0.020756 | 0 | 0 | 0 | 0.00346 |
| M300 | M26 | 0 | 0.02052 | 0 | 0 | 0.00342 |
| M300 | M279 | 0 | 0.02016 | 0 | 0 | 0.00336 |
| M232 | M230 | 0 | 0.01978 | 0 | 0 | 0.0033 |
| M188 | M139 | 0.019693 | 0 | 0 | 0 | 0.00328 |
| M80 | M105 | 0.019676 | 0 | 0 | 0 | 0.00328 |
| M80 | M229 | 0.019657 | 0 | 0 | 0 | 0.00328 |
| M65 | M112 | 0.018993 | 0 | 0 | 0 | 0.00317 |
| M63 | M253 | 0 | 0.01802 | 0 | 0 | 0.003 |
| M253 | M230 | 0 | 0.01763 | 0 | 0 | 0.00294 |
| M253 | M112 | 0.016687 | 0 | 0 | 0 | 0.00278 |
| M225 | M114 | 0.005577 | 0.01081 | 0 | 0 | 0.00273 |
| M37 | M279 | 0 | 0.0063 | 0.0095559 | 0 | 0.00264 |
| M65 | M266 | 0.015754 | 0 | 0 | 0 | 0.00263 |
| M279 | M24 | 0 | 0 | 0.015688 | 0 | 0.00261 |
| M37 | M145 | 0 | 0.01555 | 0 | 0 | 0.00259 |
| M27 | M114 | 0.004255 | 0 | 0.0111091 | 0 | 0.00256 |
| M37 | M26 | 0.003664 | 0.01127 | 0 | 0 | 0.00249 |
| M155 | M114 | 0.009244 | 0 | 6.34E-04 | 0.00147 | 0.00238 |
| M65 | M165 | 0 | 0 | 0.0131687 | 0 | 0.00219 |
| M26 | M230 | 0.003566 | 0.00913 | 0 | 0 | 0.00212 |
| M65 | M34 | 0 | 0 | 0.0116789 | 0 | 0.00195 |
| M300 | M225 | 0 | 0.01167 | 0 | 0 | 0.00194 |
| M229 | M112 | 0.011465 | 0 | 0 | 0 | 0.00191 |
| M53 | M266 | 0.011424 | 0 | 0 | 0 | 0.0019 |
| M80 | M232 | 0 | 0 | 0.0112153 | 0 | 0.00187 |
| M65 | M136 | 0.011172 | 0 | 0 | 0 | 0.00186 |
| M88 | M65 | 0.011004 | 0 | 0 | 0 | 0.00183 |
| M155 | M112 | 0.010323 | 0 | 0 | 0 | 0.00172 |
| M234 | M114 | 0.010307 | 0 | 0 | 0 | 0.00172 |
| M114 | M112 | 0.009933 | 0 | 0 | 0 | 0.00166 |
| M188 | M105 | 0.009228 | 0 | 0 | 0 | 0.00154 |
| M53 | M145 | 0 | 0 | 0.0088487 | 0 | 0.00147 |
| M65 | M300 | 0 | 0.00864 | 0 | 0 | 0.00144 |
| M80 | M27 | 0.00811 | 0 | 0 | 0 | 0.00135 |
| M88 | M225 | 0.007959 | 0 | 0 | 0 | 0.00133 |
| M63 | M266 | 0.00778 | 0 | 0 | 0 | 0.0013 |
| M266 | M145 | 0.007775 | 0 | 0 | 0 | 0.0013 |
| M266 | M155 | 0.007638 | 0 | 0 | 0 | 0.00127 |
| M80 | M26 | 0.007566 | 0 | 0 | 0 | 0.00126 |
| M37 | M253 | 0 | 0.00663 | 0 | 0 | 0.0011 |
| M37 | M136 | 0 | 0.00165 | 0.004889 | 0 | 0.00109 |
| M63 | M114 | 0 | 0 | 0.0065267 | 0 | 0.00109 |
| M145 | M105 | 0.006499 | 0 | 0 | 0 | 0.00108 |
| M190 | M65 | 0.005387 | 0 | 0 | 0 | 0.0009 |
| M37 | M252 | 0.00522 | 0 | 0 | 0 | 0.00087 |
| M80 | M252 | 0.004887 | 0 | 0 | 0 | 0.00081 |
| M252 | M225 | 0.00372 | 0 | 0 | 0 | 0.00062 |
| M83 | M37 | 0 | 0.00348 | 0 | 0 | 0.00058 |
| M252 | M230 | 0.003344 | 0 | 0 | 0 | 0.00056 |
| M80 | M24 | 0.003318 | 0 | 0 | 0 | 0.00055 |
| M37 | M34 | 0 | 0 | 0.0031913 | 0 | 0.00053 |
| M63 | M112 | 0.003183 | 0 | 0 | 0 | 0.00053 |
| M83 | M155 | 0 | 0.00265 | 0 | 0 | 0.00044 |
| M53 | M24 | 0.002295 | 0 | 0 | 0 | 0.00038 |
| M190 | M188 | 0.002279 | 0 | 0 | 0 | 0.00038 |
| M65 | M27 | 0.001949 | 0 | 0 | 0 | 0.00032 |
| M53 | M155 | 2.24E-04 | 0 | 0 | 0 | 3.7E-05 |
