# Supplementary material for: Integrated network analysis reveals potentially novel molecular mechanisms and therapeutic targets of refractory epilepsies
Source: PLoS One. 2017 Apr 7;12(4):e0174964. doi: 10.1371/journal.pone.0174964 (PMC5384674; doi:10.1371/journal.pone.0174964)
Supplement: S9 Table — Here we listed all the 193 records. (DOCX) [file pone.0174964.s009.docx]

# S9 Table. Jaccard similarity between MeSH headings. Here we listed all the 193 records.

| **MeSH heading1 (Source)** | **MeSH heading2 (Target)** | **Weight** |
| --- | --- | --- |
| Leigh Disease | Mitochondrial Encephalomyopathies | 0.273809524 |
| Classical Lissencephalies and Subcortical Band Heterotopias | Lissencephaly | 0.245614035 |
| Unverricht-Lundborg Syndrome | Myoclonic Epilepsies, Progressive | 0.220930233 |
| Epilepsy, Absence | Myoclonic Epilepsy, Juvenile | 0.2 |
| Unverricht-Lundborg Syndrome | Lafora Disease | 0.197368421 |
| MELAS Syndrome | Mitochondrial Encephalomyopathies | 0.197183099 |
| MELAS Syndrome | MERRF Syndrome | 0.196969697 |
| Leigh Disease | MELAS Syndrome | 0.194444444 |
| Myoclonic Epilepsy, Juvenile | Epilepsies, Myoclonic | 0.184331797 |
| Epilepsy, Absence | Epilepsy, Temporal Lobe | 0.179372197 |
| Mitochondrial Encephalomyopathies | MERRF Syndrome | 0.176470588 |
| Epilepsies, Myoclonic | Myoclonic Epilepsies, Progressive | 0.17472119 |
| Unverricht-Lundborg Syndrome | Epilepsies, Myoclonic | 0.173184358 |
| Epilepsy, Absence | Epilepsies, Myoclonic | 0.168888889 |
| Classical Lissencephalies and Subcortical Band Heterotopias | Malformations of Cortical Development | 0.151898734 |
| Spasms, Infantile | Classical Lissencephalies and Subcortical Band Heterotopias | 0.151515152 |
| Tuberous Sclerosis | Malformations of Cortical Development | 0.148148148 |
| Spasms, Infantile | Epilepsies, Myoclonic | 0.140776699 |
| Lissencephaly | Malformations of Cortical Development | 0.132911392 |
| Epilepsy, Absence | Epilepsy,FrontalLobe | 0.126984127 |
| Unverricht-Lundborg Syndrome | Epilepsy,FrontalLobe | 0.126582278 |
| Malformations of Cortical Development | Epilepsy, Temporal Lobe | 0.126394052 |
| Myoclonic Epilepsy, Juvenile | Epilepsy,FrontalLobe | 0.123966942 |
| Unverricht-Lundborg Syndrome | Neuronal Ceroid-Lipofuscinoses | 0.120879121 |
| Lafora Disease | Myoclonic Epilepsies, Progressive | 0.119318182 |
| Spasms, Infantile | Lissencephaly | 0.11 |
| Fragile X Syndrome | Rett Syndrome | 0.106557377 |
| Epilepsies, Myoclonic | Mitochondrial Encephalomyopathies | 0.104712042 |
| Myoclonic Epilepsy, Juvenile | Epilepsy, Temporal Lobe | 0.103004292 |
| Epilepsies, Myoclonic | Epilepsy,FrontalLobe | 0.101694915 |
| Lafora Disease | Epilepsy,FrontalLobe | 0.101449275 |
| Spasms, Infantile | Rett Syndrome | 0.1 |
| Leigh Disease | MERRF Syndrome | 0.097826087 |
| Unverricht-Lundborg Syndrome | Myoclonic Epilepsy, Juvenile | 0.094202899 |
| Lafora Disease | Epilepsy, Absence | 0.090909091 |
| Lafora Disease | Epilepsies, Myoclonic | 0.08839779 |
| Spasms, Infantile | Malformations of Cortical Development | 0.088235294 |
| Lafora Disease | Neuronal Ceroid-Lipofuscinoses | 0.085365854 |
| Epilepsies, Myoclonic | MERRF Syndrome | 0.084656085 |
| Spasms, Infantile | Tuberous Sclerosis | 0.084337349 |
| Spasms, Infantile | Epilepsy, Absence | 0.083333333 |
| Classical Lissencephalies and Subcortical Band Heterotopias | Tuberous Sclerosis | 0.076923077 |
| Spasms, Infantile | Epilepsy,FrontalLobe | 0.075471698 |
| Myoclonic Epilepsy, Juvenile | Myoclonic Epilepsies, Progressive | 0.075313808 |
| Mitochondrial Encephalomyopathies | Neuronal Ceroid-Lipofuscinoses | 0.072916667 |
| Epilepsies, Myoclonic | Neuronal Ceroid-Lipofuscinoses | 0.072164948 |
| Anti-N-Methyl-D-Aspartate Receptor Encephalitis | Landau-Kleffner Syndrome | 0.071428571 |
| Epilepsy,FrontalLobe | Epilepsy, Temporal Lobe | 0.071428571 |
| Leigh Disease | Epilepsies, Myoclonic | 0.070707071 |
| Epilepsy, Absence | Myoclonic Epilepsies, Progressive | 0.069105691 |
| Epilepsies, Myoclonic | Epilepsy, Temporal Lobe | 0.067567568 |
| Neuronal Ceroid-Lipofuscinoses | Myoclonic Epilepsies, Progressive | 0.066666667 |
| Fragile X Syndrome | Tuberous Sclerosis | 0.066225166 |
| Neuronal Ceroid-Lipofuscinoses | MERRF Syndrome | 0.065934066 |
| Epilepsy,FrontalLobe | Myoclonic Epilepsies, Progressive | 0.06557377 |
| Classical Lissencephalies and Subcortical Band Heterotopias | Rett Syndrome | 0.065420561 |
| Tuberous Sclerosis | Lissencephaly | 0.062015504 |
| Lafora Disease | MERRF Syndrome | 0.061728395 |
| Lafora Disease | Myoclonic Epilepsy, Juvenile | 0.061538462 |
| Tuberous Sclerosis | Epilepsy, Temporal Lobe | 0.06097561 |
| Spasms, Infantile | Myoclonic Epilepsy, Juvenile | 0.060240964 |
| Neuronal Ceroid-Lipofuscinoses | Landau-Kleffner Syndrome | 0.057692308 |
| Myoclonic Epilepsies, Progressive | MERRF Syndrome | 0.056701031 |
| Classical Lissencephalies and Subcortical Band Heterotopias | Fragile X Syndrome | 0.055555556 |
| Spasms, Infantile | Fragile X Syndrome | 0.0546875 |
| Spasms, Infantile | Lafora Disease | 0.054545455 |
| Epilepsy, Absence | Neuronal Ceroid-Lipofuscinoses | 0.054421769 |
| Spasms, Infantile | Epilepsy, Temporal Lobe | 0.053811659 |
| Rett Syndrome | Epilepsy, Temporal Lobe | 0.053811659 |
| Unverricht-Lundborg Syndrome | Epilepsy, Absence | 0.053691275 |
| Unverricht-Lundborg Syndrome | MERRF Syndrome | 0.053191489 |
| Rett Syndrome | Tuberous Sclerosis | 0.052631579 |
| Rett Syndrome | Malformations of Cortical Development | 0.052132701 |
| Classical Lissencephalies and Subcortical Band Heterotopias | Landau-Kleffner Syndrome | 0.05 |
| Myoclonic Epilepsies, Progressive | Epilepsy, Temporal Lobe | 0.049833887 |
| Epilepsies, Myoclonic | MELAS Syndrome | 0.049723757 |
| Epilepsies, Myoclonic | Rett Syndrome | 0.049107143 |
| Epilepsy,FrontalLobe | Neuronal Ceroid-Lipofuscinoses | 0.048192771 |
| Epilepsy, Absence | Rett Syndrome | 0.045977011 |
| Mitochondrial Encephalomyopathies | Myoclonic Epilepsies, Progressive | 0.044554455 |
| Classical Lissencephalies and Subcortical Band Heterotopias | Anti-N-Methyl-D-Aspartate Receptor Encephalitis | 0.044444444 |
| Lafora Disease | Tuberous Sclerosis | 0.044117647 |
| Fragile X Syndrome | Malformations of Cortical Development | 0.041025641 |
| Unverricht-Lundborg Syndrome | Spasms, Infantile | 0.040322581 |
| Spasms, Infantile | Leigh Disease | 0.03968254 |
| Unverricht-Lundborg Syndrome | Mitochondrial Encephalomyopathies | 0.03960396 |
| Unverricht-Lundborg Syndrome | Fragile X Syndrome | 0.037735849 |
| Rett Syndrome | Lissencephaly | 0.037383178 |
| Epilepsy, Absence | Malformations of Cortical Development | 0.037344398 |
| Unverricht-Lundborg Syndrome | Landau-Kleffner Syndrome | 0.036363636 |
| Alexander Disease | Tuberous Sclerosis | 0.035714286 |
| Spasms, Infantile | Myoclonic Epilepsies, Progressive | 0.035242291 |
| Neuronal Ceroid-Lipofuscinoses | Epilepsy, Temporal Lobe | 0.034825871 |
| Myoclonic Epilepsy, Juvenile | Neuronal Ceroid-Lipofuscinoses | 0.034722222 |
| Epilepsy,FrontalLobe | Malformations of Cortical Development | 0.034090909 |
| Myoclonic Epilepsy, Juvenile | Malformations of Cortical Development | 0.033898305 |
| Lafora Disease | Mitochondrial Encephalomyopathies | 0.033707865 |
| Fragile X Syndrome | Epilepsies, Myoclonic | 0.033492823 |
| Fragile X Syndrome | Epilepsy, Temporal Lobe | 0.033492823 |
| Alexander Disease | Neuronal Ceroid-Lipofuscinoses | 0.032786885 |
| Alexander Disease | Malformations of Cortical Development | 0.032679739 |
| Unverricht-Lundborg Syndrome | Rett Syndrome | 0.032 |
| Classical Lissencephalies and Subcortical Band Heterotopias | Epilepsy, Temporal Lobe | 0.031746032 |
| Epilepsy, Absence | Fragile X Syndrome | 0.03164557 |
| Fragile X Syndrome | Anti-N-Methyl-D-Aspartate Receptor Encephalitis | 0.03030303 |
| Unverricht-Lundborg Syndrome | Epilepsy, Temporal Lobe | 0.029411765 |
| Epilepsy, Absence | Classical Lissencephalies and Subcortical Band Heterotopias | 0.028985507 |
| Leigh Disease | Fragile X Syndrome | 0.027522936 |
| Epilepsies, Myoclonic | Malformations of Cortical Development | 0.027118644 |
| Lafora Disease | Classical Lissencephalies and Subcortical Band Heterotopias | 0.027027027 |
| Lafora Disease | Rett Syndrome | 0.026548673 |
| Anti-N-Methyl-D-Aspartate Receptor Encephalitis | Malformations of Cortical Development | 0.026490066 |
| Lissencephaly | Landau-Kleffner Syndrome | 0.026315789 |
| Lafora Disease | Epilepsy, Temporal Lobe | 0.026041667 |
| Epilepsy, Absence | Tuberous Sclerosis | 0.024630542 |
| Epilepsy,FrontalLobe | MERRF Syndrome | 0.024390244 |
| Leigh Disease | Myoclonic Epilepsies, Progressive | 0.024154589 |
| Fragile X Syndrome | Myoclonic Epilepsies, Progressive | 0.023696682 |
| Classical Lissencephalies and Subcortical Band Heterotopias | Neuronal Ceroid-Lipofuscinoses | 0.023529412 |
| Leigh Disease | Lissencephaly | 0.023255814 |
| Myoclonic Epilepsy, Juvenile | Rett Syndrome | 0.023255814 |
| Myoclonic Epilepsy, Juvenile | Lissencephaly | 0.023076923 |
| Myoclonic Epilepsy, Juvenile | Classical Lissencephalies and Subcortical Band Heterotopias | 0.022556391 |
| Leigh Disease | Classical Lissencephalies and Subcortical Band Heterotopias | 0.02247191 |
| Fragile X Syndrome | Lissencephaly | 0.022222222 |
| Alexander Disease | Lissencephaly | 0.02173913 |
| Anti-N-Methyl-D-Aspartate Receptor Encephalitis | Epilepsy,FrontalLobe | 0.02173913 |
| Rett Syndrome | Myoclonic Epilepsies, Progressive | 0.02173913 |
| MELAS Syndrome | Myoclonic Epilepsies, Progressive | 0.021505376 |
| Lissencephaly | Epilepsy, Temporal Lobe | 0.021276596 |
| Classical Lissencephalies and Subcortical Band Heterotopias | Epilepsies, Myoclonic | 0.020942408 |
| Neuronal Ceroid-Lipofuscinoses | Malformations of Cortical Development | 0.020942408 |
| Alexander Disease | Classical Lissencephalies and Subcortical Band Heterotopias | 0.020408163 |
| Leigh Disease | Neuronal Ceroid-Lipofuscinoses | 0.019607843 |
| MERRF Syndrome | Landau-Kleffner Syndrome | 0.019607843 |
| Epilepsies, Myoclonic | Tuberous Sclerosis | 0.01953125 |
| Tuberous Sclerosis | Myoclonic Epilepsies, Progressive | 0.01953125 |
| Anti-N-Methyl-D-Aspartate Receptor Encephalitis | Epilepsy, Temporal Lobe | 0.018181818 |
| Alexander Disease | Epilepsy, Temporal Lobe | 0.017857143 |
| Mitochondrial Encephalomyopathies | Landau-Kleffner Syndrome | 0.01754386 |
| Alexander Disease | MERRF Syndrome | 0.016949153 |
| Anti-N-Methyl-D-Aspartate Receptor Encephalitis | Neuronal Ceroid-Lipofuscinoses | 0.016949153 |
| Myoclonic Epilepsies, Progressive | Malformations of Cortical Development | 0.016778523 |
| Spasms, Infantile | MERRF Syndrome | 0.016393443 |
| Fragile X Syndrome | Landau-Kleffner Syndrome | 0.016129032 |
| Rett Syndrome | Neuronal Ceroid-Lipofuscinoses | 0.016 |
| Spasms, Infantile | Mitochondrial Encephalomyopathies | 0.015625 |
| Leigh Disease | Rett Syndrome | 0.015503876 |
| MELAS Syndrome | Lissencephaly | 0.015384615 |
| Epilepsy, Absence | Lissencephaly | 0.01459854 |
| Epilepsy,FrontalLobe | Tuberous Sclerosis | 0.014492754 |
| Alexander Disease | Fragile X Syndrome | 0.014285714 |
| Epilepsy,FrontalLobe | Lissencephaly | 0.014285714 |
| Myoclonic Epilepsy, Juvenile | MERRF Syndrome | 0.013888889 |
| Classical Lissencephalies and Subcortical Band Heterotopias | Epilepsy,FrontalLobe | 0.01369863 |
| Landau-Kleffner Syndrome | Malformations of Cortical Development | 0.013513514 |
| Myoclonic Epilepsy, Juvenile | Fragile X Syndrome | 0.012903226 |
| Lissencephaly | MERRF Syndrome | 0.0125 |
| Epilepsies, Myoclonic | Landau-Kleffner Syndrome | 0.01242236 |
| Myoclonic Epilepsies, Progressive | Landau-Kleffner Syndrome | 0.01242236 |
| MELAS Syndrome | Neuronal Ceroid-Lipofuscinoses | 0.012345679 |
| Unverricht-Lundborg Syndrome | MELAS Syndrome | 0.012048193 |
| Classical Lissencephalies and Subcortical Band Heterotopias | MERRF Syndrome | 0.012048193 |
| Lissencephaly | Neuronal Ceroid-Lipofuscinoses | 0.012048193 |
| Mitochondrial Encephalomyopathies | Lissencephaly | 0.011627907 |
| Unverricht-Lundborg Syndrome | Classical Lissencephalies and Subcortical Band Heterotopias | 0.011363636 |
| Spasms, Infantile | Alexander Disease | 0.011235955 |
| Fragile X Syndrome | Epilepsy,FrontalLobe | 0.010638298 |
| Epilepsies, Myoclonic | Lissencephaly | 0.010526316 |
| Lafora Disease | Fragile X Syndrome | 0.010416667 |
| Leigh Disease | Malformations of Cortical Development | 0.010152284 |
| Myoclonic Epilepsy, Juvenile | Landau-Kleffner Syndrome | 0.009708738 |
| Fragile X Syndrome | MERRF Syndrome | 0.009615385 |
| Fragile X Syndrome | Neuronal Ceroid-Lipofuscinoses | 0.009345794 |
| Spasms, Infantile | MELAS Syndrome | 0.009259259 |
| Epilepsy, Absence | Landau-Kleffner Syndrome | 0.009174312 |
| Epilepsy,FrontalLobe | Rett Syndrome | 0.008849558 |
| Epilepsy, Absence | Alexander Disease | 0.008547009 |
| Rett Syndrome | MERRF Syndrome | 0.008130081 |
| Spasms, Infantile | Neuronal Ceroid-Lipofuscinoses | 0.007936508 |
| Tuberous Sclerosis | MERRF Syndrome | 0.006711409 |
| Epilepsy, Absence | MERRF Syndrome | 0.006622517 |
| Myoclonic Epilepsy, Juvenile | Mitochondrial Encephalomyopathies | 0.006622517 |
| Tuberous Sclerosis | Neuronal Ceroid-Lipofuscinoses | 0.006578947 |
| Unverricht-Lundborg Syndrome | Tuberous Sclerosis | 0.006493506 |
| Leigh Disease | Tuberous Sclerosis | 0.006410256 |
| Epilepsy, Absence | Mitochondrial Encephalomyopathies | 0.006369427 |
| Lafora Disease | Malformations of Cortical Development | 0.005464481 |
| Lissencephaly | Myoclonic Epilepsies, Progressive | 0.005235602 |
| Classical Lissencephalies and Subcortical Band Heterotopias | Myoclonic Epilepsies, Progressive | 0.005154639 |
| Unverricht-Lundborg Syndrome | Malformations of Cortical Development | 0.005102041 |
| Myoclonic Epilepsy, Juvenile | Tuberous Sclerosis | 0.004975124 |
| MERRF Syndrome | Epilepsy, Temporal Lobe | 0.004901961 |
