# Supplementary material for: Integrated network analysis reveals potentially novel molecular mechanisms and therapeutic targets of refractory epilepsies
Source: PLoS One. 2017 Apr 7;12(4):e0174964. doi: 10.1371/journal.pone.0174964 (PMC5384674; doi:10.1371/journal.pone.0174964)
Supplement: S10 Table — (DOCX) [file pone.0174964.s010.docx]

**S10 Table. Drugs and targets distributed in modules.**

| **Module** | **Number of proteins** | **Number of AEDs targets** | **Number of AEDs** |
| --- | --- | --- | --- |
| 155 | 141 | 27 | 48 |
| 65 | 184 | 24 | 20 |
| 147 | 159 | 6 | 3 |
| 227 | 106 | 5 | 9 |
| 48 | 204 | 4 | 3 |
| 144 | 170 | 4 | 2 |
| 146 | 149 | 4 | 3 |
| 188 | 43 | 4 | 10 |
| 95 | 204 | 3 | 21 |
| 294 | 7 | 3 | 11 |
| 6 | 77 | 2 | 1 |
| 49 | 200 | 2 | 1 |
| 69 | 96 | 2 | 4 |
| 70 | 131 | 2 | 1 |
| 11 | 178 | 1 | 1 |
| 43 | 185 | 1 | 1 |
| 55 | 131 | 1 | 1 |
| 90 | 17 | 1 | 2 |
| 105 | 6 | 1 | 2 |
| 126 | 63 | 1 | 1 |
| 127 | 95 | 1 | 1 |
| 162 | 9 | 1 | 1 |
| 193 | 5 | 1 | 1 |
| 205 | 142 | 1 | 1 |
| 214 | 105 | 1 | 1 |
| 240 | 11 | 1 | 4 |
| 254 | 30 | 1 | 1 |
